# Amyotrophic lateral sclerosis and frontotemporal dementia mutation reduces endothelial TDP-43 and causes blood-brain barrier defects

**DOI:** 10.1126/sciadv.ads0505

**Published:** 2025-04-16

**Authors:** Ashok Cheemala, Amy L. Kimble, Emily N. Burrage, Stephen B. Helming, Jordan D. Tyburski, Nathan K. Leclair, Omar M. Omar, Aamir R. Zuberi, Melissa Murphy, Evan R. Jellison, Bo Reese, Xiangyou Hu, Cathleen M. Lutz, Riqiang Yan, Patrick A. Murphy

**Affiliations:** ^1^ Center for Vascular Biology, University of Connecticut School of Medicine, Farmington, CT, USA.; ^2^ MD/PhD Program, University of Connecticut School of Medicine, Farmington, CT, USA.; ^3^ Rare Disease Translational Center and Technology Evaluation and Development Laboratory, The Jackson Laboratory, Bar Harbor, ME, USA.; ^4^ Department of Immunology, University of Connecticut School of Medicine, Farmington, CT, USA.; ^5^ Center for Genome Innovation, University of Connecticut, Storrs, CT, USA.; ^6^ Department of Neuroscience, University of Connecticut School of Medicine, Farmington, CT, USA.

## Abstract

Mutations in the *TARDBP* gene encoding TDP-43 protein are linked to loss of function in neurons and familial frontotemporal dementia (FTD) and amyotrophic lateral sclerosis (ALS). We recently identified reduced nuclear TDP-43 in capillary endothelial cells (ECs) of donors with ALS-FTD. Because blood-brain barrier (BBB) permeability increases in ALS-FTD, we postulated that reduced nuclear TDP-43 in ECs might contribute. Here, we show that nuclear TDP-43 is reduced in ECs of mice with an ALS-FTD–associated mutation in TDP-43 (*Tardbp^G348C^*) and that this leads to cell-autonomous loss of junctional complexes and BBB integrity. Targeted excision of TDP-43 in brain ECs recapitulates BBB defects and loss of junctional complexes and ultimately leads to fibrin deposition, gliosis, phospho-Tau accumulation, and impaired memory and social interaction. Transcriptional changes in TDP-43–deficient ECs resemble diseased brain ECs. These data show that nuclear loss of TDP-43 in brain ECs disrupts the BBB and causes hallmarks of FTD.

## Teaser

ALS-FTD–associated TDP-43 point mutation in endothelial cells leads to BBB loss and is replicated by gene deletion.

## INTRODUCTION

Loss of nuclear TDP-43 (TAR DNA-binding protein 43) is a common feature in a wide range of neurodegenerative diseases (*1*). These include Alzheimer’s disease (AD), limbic-predominant age-related TDP-43 encephalopathy, and amyotrophic lateral sclerosis and frontotemporal dementia (ALS-FTD). Across these diseases, a common feature is aggregation of ubiquitinated TDP-43 in the cytosol and nuclear loss of TDP-43 in neurons. The identification of familial FTD mutations in TDP-43 that exacerbate this process highlights TDP-43 dysfunction as a driver in disease progression. Mechanistically, the reduced nuclear levels of TDP-43 are associated with impaired nuclear splicing functions. In a dose-dependent manner, the loss of nuclear TDP-43 results in the aberrant inclusion of exonic junctions into transcripts, often leading to transcript destabilization and degeneration through nonsense-mediated mRNA decay (*2*–*5*). In neurons, the loss of specific transcripts alters the expression of proteins critical for axonal projection, which is thought to contribute to the progression of motor neuron deficits in ALS. In addition to its prominent effect on neurons, TDP-43 dysfunction is observed in various cell types, including fibroblasts isolated from donors with ALS-FTD (*6*), pancreatic islet cells (*7*), and astrocytes (*8*–*10*), suggesting the possibility that its dysfunction in other cell types may also contribute to disease progression. Moreover, as ALS-FTD represents a broad spectrum of diseases with similar underlying mutations (*11*, *12*), the specific cell types most affected may be critical in determining disease presentation.

Early in the course of neurodegenerative diseases, increased flux across the blood-brain barrier (BBB) is detected by gadolinium-based contrast agents in magnetic resonance imaging, a finding supported by elevated cerebrospinal fluid albumin levels (*13*–*15*). BBB leakage alone can exacerbate neurodegenerative changes in animal models of superoxide dismutase type 1–driven ALS (*16*, *17*). In a mouse model of ALS-FTD with *Tardbp^Q331K^* homozygous mutation, alterations in the vascular compartment precede microglial activation, neuronal loss, and behavioral symptoms (*18*), suggesting that these conditions may begin with a leaky BBB. While not all the subsequent consequences of BBB leakage are fully understood, fibrin deposition has been linked to reactive changes in brain microglia (*19*). The BBB is part of a complex neurovascular unit comprising endothelial cells (ECs) lining the lumen of vessels, an underlying basement membrane, associated pericytes, astrocytes, and perivascular fibroblasts. Although each of these components contributes to the barrier, it is the ECs that provide the functional barrier through tight junctions and adapter proteins (e.g., claudin-5 and ZO-1), basal adherens junctions (e.g., VE-cadherin), tight regulation of general endocytic pathways (*20*), as well as specific transporters and efflux pathways [e.g., P-glycoprotein (*ABCB1*) and breast cancer resistance protein (*BCRP*)] (*21*, *22*). While TDP-43 is highly expressed in the endothelium, the consequence of nuclear TDP-43 loss of function within the endothelium on BBB is unknown.

In a parallel manuscript, we identified loss of TDP-43 nuclear protein in ~40% of capillaries in AD and ALS-FTD (*23*). This loss coincided with markers of reduced Wnt signaling and increased nuclear factor κB (NF-κB) signaling. Mechanistically, Wnt signaling is essential for the initiation and maintenance of BBB properties (*21*), while NF-κB signaling is known to be increased following a wide range of pathological insults and results in BBB dysfunction. Together, our results led us to hypothesize that the loss of nuclear TDP-43 in ECs could contribute to BBB defects in the progression of neurodegeneration. Here, we test this hypothesis, using a knock-in mouse model of ALS-FTD and pan-endothelial–and brain-endothelial–specific knockouts (KOs) of TDP-43.

## RESULTS

### *Tardbp^G348C/+^* model of ALS-FTD exhibits brain barrier leak in vivo

**Fig. 1.**
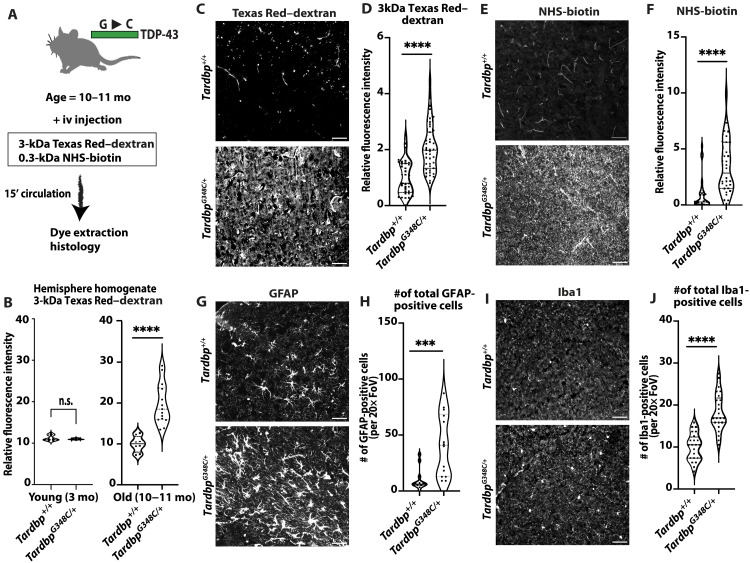
BBB disruption astrogliosis and microgliosis in *Tardbp^G348C/+^* mice. (**A**) A schematic illustration of the assay for measuring BBB permeability. mo, months; iv, intravenous; 15′ circulation, 15 min of circulation. (**B**) The quantification process involved homogenizing brain tissue samples from 3-month-old wild-type (*n* = 3) and *Tardbp^G348C/+^* mice (*n* = 3) and 10- to 11-month-old wild-type *Tardbp^+/+^* mice (*n* = 8) and their heterozygous littermates, *Tardbp^G348C/+^* mice (*n* = 15), followed by measuring fluorescence intensity at 590 nm. n.s., not significant. Representative images of (**C**) 3-kDa Texas Red–dextran leakage, (**E**) NHS-biotin, (**G**) glial fibrillary acidic protein (GFAP) staining of astrocytes, and (**I**) Iba1 staining of microglia in the mouse brain cortex reveal consistent results across *Tardbp^+/+^* mice (*n* = 3) and *Tardbp^G348C/+^* mice (*n* = 3). [(B), **D**, and **F**] Quantification of data, with each data point representing the fluorescence image intensity in one image. (**H** and **J**) Field of view (FoV) is 0.16 mm^2^. Quantification of data with each data point representing the number of activated cells in an image, with multiple images per mouse. Scale bars, 50 μm. Data are presented as means ± SEM. Statistical analysis was conducted using an unpaired Mann-Whitney test, with significance levels indicated as follows: ***P < 0.001; ****P < 0.0001.

A mouse model of a frequent familial ALS-FTD G348C mutation (*24*) was generated by CRISPR knock-in to the endogenous locus. The mice were viable and exhibited a nearly exact Mendelian ratio from het × het intercrosses (55 wild type, 110 *Tardbp^G348C/+^*, and 52 *Tardbp^G348C/G348C^*). To examine brain barrier function in young (3-month) and older (10- to 11-month) *Tardbp^G348C/+^* mice, we injected tomato lectin, 3-kDa Texas Red–dextran, and 0.3-kDa NHS-sulfo-biotin into the circulation of the mice and their littermate controls. After 15 min of circulation, mice were euthanized, and the brain was extracted. The right posterior quadrant containing the cerebellum, midbrain, and cortex was used for dye extraction, and analysis (Fig. 1A). We found that old, and not young, *Tardbp^G348C/+^* mice exhibited increased dye leak (Fig. 1B). To examine leakage within specific brain vascular beds, we examined tissue sections from these mice and found increased and diffuse staining from both larger 3-kDa Texas Red–dextran and the smaller 0.3-kDa NHS-sulfo-biotin, detected by streptavidin 647 (Fig. 1, C to F, and fig. S1, A to D). We did not observe obvious focal leak. Midbrain and cortex appeared to be similarly affected, suggesting that increased staining resulted from systemic vascular defects (fig. S1). Although lectin signal appeared to be reduced, this likely reflects lower lectin binding to the endothelium, as lipophilic DiI perfusion labeling of the vasculature indicated similar densities and sorting ECs from the mice indicated lower per cell levels of lectin staining (fig. S2). Reduced lectin binding may indicate a loss of glycocalyx, a protective layer on the endothelium, which can become damaged in states of chronic endothelial activation (*25*).

As BBB dysfunction is linked to astrogliosis and microgliosis (*22*, *26*, *27*), we examined astrocytes using glial fibrillary acidic protein (GFAP) and microglia using Iba1 and found significantly increased numbers in the *Tardbp^G348C/+^* mice (Fig. 1, G to J, and fig. S3, A to D). We also observed increased levels of phospho-Tau staining in neurons, an indication of Tau dysfunction and neuronal dysfunction in neurodegeneration disease (fig. S4). Strong activation of the endothelium is associated with increased expression of Icam1 and recruitment of CD45^high^ hematopoietic cells from the circulation across the endothelium and into tissues. Although we observed a trend toward increased Icam1 expression in sorted brain ECs, we did not observe an increase in CD45^high^ in brain tissue (fig. S5). Thus, the data support an age-dependent disruption of the BBB and reactive gliosis and indications of neuronal damage, with a progression that appears similar to a *Tardbp^Q331V/Q331V^* model of ALS-FTD (*18*).

**Fig. 2.**
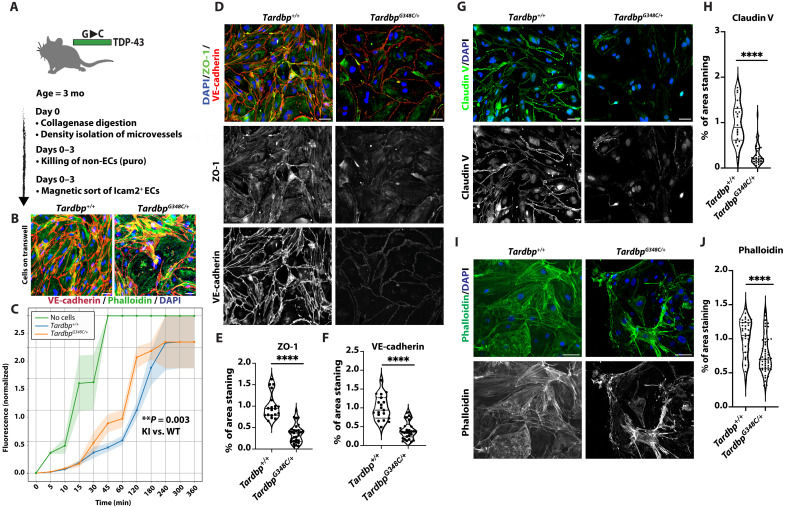
Defects in permeability and cell junctions in *Tardbp^G348C/+^* ECs. (**A**) Schematic illustration of the isolation and purification of ECs. (**B**) Immunofluorescence image of cells on transwell filter at endpoint of in vitro permeability experiment. Color of phalloidin is preferentially enhanced in G348C image to show cell coverage. Scale bars, 50 μm. (**C**) Passage of 10-kDa fluorescein isothiocyanate (FITC)–dextran dye across confluent monolayers of ECs isolated from *Tardbp^+/+^* (*n* = 6) and *Tardbp^G348C/+^* (*n* = 6), where each replicate indicates cells isolated from a separate mouse with age matched controls. Dye leak is normalized to wild type (WT) at 120 min. KI, knock-in. Statistical analysis was conducted using two-way analysis of variance (ANOVA) with repeated measurements. (**D**, **G**, and **I**) Representative images of mouse brain ECs isolated from 3-month-old wild-type *Tardbp^+/+^* (*n* = 3) and their heterozygous littermates, *Tardbp^G348C/+^* mice (*n* = 3), immunostained with antibodies to the indicated proteins. (**E**, **F**, **H**, and **J**) Quantification of data, with each data point representing the fluorescence image intensity in one image, multiple images taken of cells from each mouse. Scale bars, 50 μm. Data are presented as means ± SEM. Statistical analysis was conducted using an unpaired Mann-Whitney test, with significance levels indicated as follows: ****P ≤ 0.0001.

ECs of the brain are highly specialized to provide barrier function through the expression of tight junction proteins (claudins, occludins, and junctional adhesion molecules), tight regulation of endocytic pathways, and expression of efflux transporters, including breast cancer resistance protein (Bcrp/Abcg2) and ATP (adenosine 5′-triphosphate)–binding cassette, subfamily B (Abcb1) (*21*). Therefore, we asked whether barrier dysfunction in *Tardbp^G348C/+^* could be due to effects on the endothelium. We purified brain ECs from *Tardbp^G348C/+^* mice and littermate controls. Although we obtained similar numbers of each, we noted impaired expansion of *Tardbp^G348C/+^* cells in vitro (fig. S6), indicating cell intrinsic defects. To examine their ability to produce a barrier, we seeded confluent transwell filters with the same number of cells. We found that *Tardbp^G348C/+^* ECs exhibited increased permeability of 10-kDa fluorescein isothiocyanate (FITC)–dextran dye, relative to cells derived from littermate controls (Fig. 2, A to C). The finding was robust and was replicated across six independent and age-matched mice of each genotype. We confirmed confluence by immunostaining of transwell filters for VE-cadherin, phalloidin, and 4′,6-diamidino-2-phenylindole (DAPI) after completion of the experiment (Fig. 2B).

We noted a reduction in cell junction protein intensity and phalloidin staining on the transwell filters of *Tardbp^G348C/+^* cells. To examine this further, we stained junctional proteins associated with barrier function and observed a reduction in VE-cadherin, claudin-5, and ZO-1 (Fig. 2, D to H). The cytoskeleton is critical for the establishment of cell-cell junctions (*28*), and staining of actin by phalloidin indicated significant alterations in cellular actin (Fig. 2, I and J). Furthermore, immunostaining for tubulin and paxillin indicated large changes in both microtubules and focal adhesions, with distinct changes occurring in *Tardbp^G348C/+^* cells and worsening in *Tardbp^G348C/G348C^* cells (fig. S7).

Thus, a single ALS-FTD–associated amino acid substitution in heterozygous *Tardbp^G348C/+^* mice is sufficient to cause cell-autonomous defects in ECs, including reduced expansion and increased leak across the BBB. As isolated ECs exhibit defective barrier function and cell-cell junctions, the data suggest that at least some of this effect is due to dysfunction in endothelial junctions and the cytoskeleton.

### Nuclear levels of TDP-43 are reduced in the ECs of *Tardbp^G348C/+^* mice

**Fig. 3.**
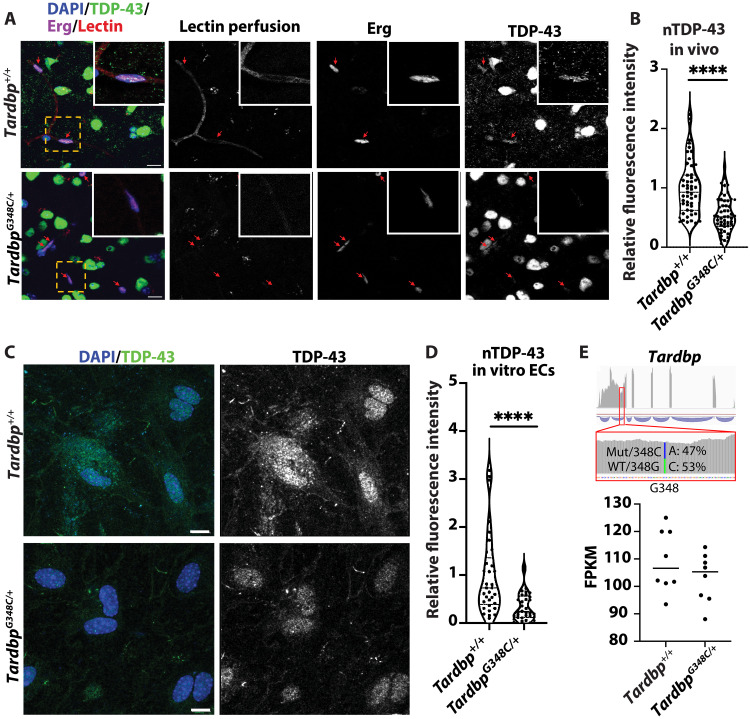
Reduced nuclear TDP-43 in ECs of *Tardbp^G348C/+^* mice. (**A**) Representative confocal images of mouse brain frontal cortex sections from 10-month-old wild-type *Tardbp^+/+^* (*n* = 6) and their heterozygous littermates, *Tardbp^G348C/+^* mice (*n* = 6). Shown are representative low-magnification and high-magnification immunofluorescence images for the indicated markers. Each red arrow indicates vascular endothelial nuclear TDP-43 immunostaining. (**B**) Quantification of TDP-43; each data point represents one vascular EC nucleus. (**C**) Representative confocal images of mouse brain ECs isolated from 3-month-old wild-type *Tardbp^+/+^* (*n* = 6) and their heterozygous littermates, *Tardbp^G348C/+^* mice (*n* = 6), immunostained with antibodies against endogenous TDP-43 (green) and DAPI (blue). (**D**) Quantification of nuclear TDP-43 levels in isolated ECs, confirming a reduction in TDP-43 in *Tardbp^G348C/+^* mice; each data point represents one EC nucleus. (**E**) RNA-sequencing analysis of ECs derived from *Tardbp^G348C/+^* mice and littermate controls showed no significant differences in mRNA expression. Phased analysis of transcripts derived from the mutated G348C allele and the wild-type allele in the same cells revealed no differences in mRNA transcript levels. Data are presented as means ± SEM. Statistical analysis was conducted using an unpaired Mann-Whitney test, ****P ≤ 0.0001. [(A) and (C)] FPKM, fragments per kilobase of exon per million mapped fragments. Scale bars, 50 μm.

ALS-FTD–associated mutations in TDP-43 are associated with cytoplasmic accumulation and reduced nuclear levels of protein (*1*). To determine whether the loss of nuclear TDP-43 occurs in the brain endothelium of *Tardbp^G348C/+^* mice, we stained tissue sections for the protein and examined colocalization with DAPI^+^ nuclei in lectin-perfused vessels in the cerebral cortex. Although detecting aggregation in thin ECs in vivo is difficult, we found that nuclear TDP-43 levels were reduced relative to littermate controls (Fig. 3, A and B). To more broadly examine this, we also performed flow cytometry of isolated NeuN^+^ neuronal nuclei and Erg^+^ endothelial nuclei. NeuN antibody binds an Rbfox3 epitope specific to neurons, and Erg antibody (ETS-related gene) binds the endothelial specific transcription factor. We found that overall expression of TDP-43 in endothelial nuclei is lower than neuronal nuclei, supporting similar observations in tissue staining and that TDP-43 was reduced in both neuronal and endothelial nuclei (fig. S8). We noted that the reduction in nuclear TDP-43 was more pronounced in the Erg^+^ endothelial nuclei than in NeuN^+^ neuronal nuclei (fig. S8). To further confirm this, we examined isolated ECs from the cortex and stained them in culture for TDP-43, revealing that the reduction in nuclear TDP-43 is preserved in vitro (Fig. 3, C and D).

We then asked whether reduced nuclear TDP-43 could be a result of reduced TDP-43 mRNA in the cells. RNA-sequencing analysis in vivo and in vitro revealed no significant differences in mRNA expression in ECs derived from *Tardbp^G348C/+^* mice versus their littermate controls (Fig. 3E). Furthermore, phased analysis of the transcripts derived from the mutated G348C allele versus the wild-type allele in the same cells showed no differences in mRNA transcript (Fig 3E, top).

Together, these data indicate the introduction of the G348C mutation in *Tardbp^G348C/+^* mice leads to a loss in nuclear levels of the protein. This is not due to a significant change in total cellular mRNA from the mutant allele.

### Endothelial deletion of *Tardbp* causes systemic EC activation

**Fig. 4.**
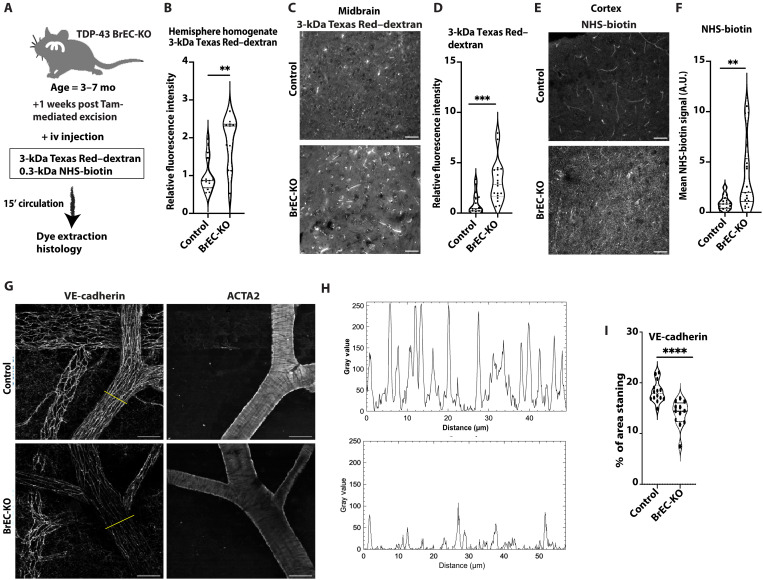
BBB disruption in *Tardbp* BrEC-KO mice. (**A**) Schematic representation of the assay for measuring BBB permeability. (**B**) Measurement of 3-kDa Texas Red–dextran in homogenized brain tissue 1 week after tamoxifen (Tam) treatment of BrEC-KO mice (*n* = 11) and littermate controls (*n* = 12). **P < 0.0034. (**C** and **E**) Representative images of (C) 3-kDa Texas Red–dextran leakage and (E) NHS-biotin in the cortex of 3- to 7-month-old mice (*n* = 3 BrEC-KO and *n* = 3 littermate controls). (**D** and **F**) Quantification signal, with each data point representing the fluorescence image intensity from one image, multiple images per mouse. A.U., arbitrary units. (**G**) Whole-mount immunostaining for VE-cadherin and α–smooth muscle actin (*n* = 12 arteries in 3 BrECKO mice and *n* = 14 arteries in 4 controls), with (**H**) quantitation of signal intensity in the artery, across the yellow line in (G). (**I**) Quantitation by animal of threshold VE-cadherin staining in arteries. [(C), (E), and (G)] Scale bars, 50 μm. Data are presented as means ± SEM. Statistical analysis was conducted using an unpaired Mann-Whitney test, with significance levels indicated as follows: **P < 0.01; ***P < 0.001; ****P ≤ 0.0001.

The reduction in nuclear TDP-43 in *Tardbp^G348C/+^* mice and the cytoskeletal and junctional defects in ECs isolated from these mice suggested that ECs may be sensitive to reduced TDP-43 levels, potentially contributing to BBB dysfunction. To specifically address this, we generated *Cdh5(PAC)CreERT2; Tardbp^ff^* (EC-KO) mice and *Tardbp^ff^* littermate controls. Consistent with a recent report (*29*), postnatal excision of the floxed gene in the endothelium caused morbidity within 3 to 4 weeks (fig. S9A). This is true whether the gene is excised in the early postnatal period, as recently reported, or in adult mice, as we report here. The mice exhibited a range of vascular defects consistent with systemic EC activation. These included a leak of Evans blue dye (fig. S9B), reduced platelet counts in circulation (Fig. 4C), impaired ejection fraction in the heart (fig. S9, D and E), and increased fibrosis (fig. S9F).

While pan-endothelial deletion of *Tardbp* data indicated a specific requirement in the endothelium and recapitulated endothelial permeability observed in *Tardbp^G348C/+^* mice, the rapid development of systemic vascular dysfunction precluded further analysis of brain-specific functions. Therefore, we generated *Slco1c1(BAC)iCreERT2; Tardbp^ff^* (BrEC-KO) mice and *Tardbp^ff^* littermate controls for deletion of *Tardbp* specifically within the brain endothelium. In these mice, CreER is driven by a transporter promoter with high specificity to the brain endothelium and epithelial cells of the choroid plexus (*30*). As expected, we observed Cre activity in brain ECs but no deletion in other cell types of the brain (fig. S10) and confirmed a reduction in endothelial but not neuronal TDP-43 by flow cytometry (fig. S11). We found that, within a week of tamoxifen-induced gene excision in adult mice, injection of 3-kDa Texas Red–dextran accumulated at higher levels in total brain extract after intravascular injection, which suggested an increased leak across the BBB (Fig. 4, A and B, and fig. S12, A and B). Although dye leak could theoretically arise from leakage across the choroid plexus, the pattern of leak of both 3-kDa Texas Red–dextran and 0.3-kDa NHS-sulfo-biotin within the cortex was pervasive, and not localized to the ventricle-adjacent regions (Fig. 4, C to F, and fig. S12, A and B), as would be expected of choroid plexus leak. Moreover, we observed a loss-of-junction VE-cadherin protein in BrEC-KO mice in vivo, which closely approximated the loss of VE-cadherin in *Tardbp^G348C/+^* ECs (Fig. 4, G to I). This reduction was most clearly appreciated in smooth muscle actin–positive arteries and arterioles and less in veins.

Also similar to *Tardbp^G348C/+^* mice, we observed reduced lectin staining intensity in brain vasculature of BrEC-KO mice and reduced staining intensity in sorted CD45^neg^/CD31^+^/Icam2^+^ ECs (fig. S13). Increased levels of Icam1 (fig. S5) and of basement membrane proteins fibronectin and collagen IV mirrored those in the *Tardbp^G348C/+^* mice and may be a response to BBB dysfunction (figs. S14 and S15). Similar changes in basement membrane proteins have been observed in some studies of neurodegenerative diseases, including AD and ALS-FTD (*31*). In general, these changes were all more severe in BrEC-KO mice than in *Tardbp^G348C/+^* mice, perhaps due to the difference between a partial and complete loss of TDP-43 function. Therefore, two independent means of endothelial *Tardbp* excision confirm a critical requirement in the endothelium and that specific loss within brain endothelium recapitulates barrier defects observed in *Tardbp^G348C/+^* mice.

### RNA sequencing reveals common TDP-43–sensitive pathways in the endothelium

**Fig. 5.**
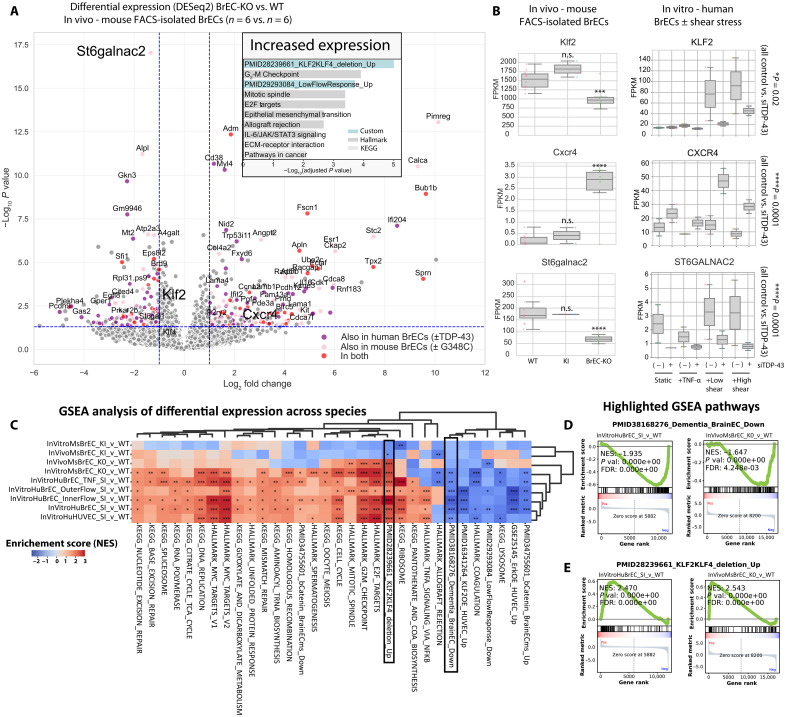
Core BBB pathways affected by loss of TDP-43. Analysis of differential gene expression in acutely isolated ECs from BrEC-KO mice and littermate controls. (**A**) Volcano plot showing differential gene expression by DESeq2. Colors indicate that gene expression changes are consistent in direction between datasets. Inset shows Enrichr analysis of up-regulated genes. Large font points are shown for each dataset in (**B**), along with changes in expression in human primary brain ECs with and without siTDP-43 in the indicated culture conditions [static culture, static culture with TNF-α (30 ng/ml), or rotational culture and high shear or low shear, from inner or outer region of the well]. IL-6/JAK/STAT3, interleukin-6/Janus kinase/signal transducer and activator of transcription 3; ECM, extracellular matrix; FACS, fluorescence-activated cell sorting. (**C**) Pathway analysis by GSEApy, of the most consistently regulated transcripts in acutely isolated brain ECs from BrECKO and KI mice, cultured ECs from the same mice, or primary human brain ECs with suppression of TDP-43 by small interfering RNA (siRNA). (**D** and **E**) Example gene set enrichment analysis (GSEA) plots for top scoring pathways. Heatmap and clustering (UPGMA, unweighted pair group method with arithmetic mean algorithm) shows the most consistently affected Kyoto Encyclopedia of Genes and Genomes (KEGG) and Hallmark pathways (human to mouse liftOver by BioMart) and custom pathways. Details on the gene sets used for GSEA and the RNA-sequencing samples are contained in table S1. False discovery rate (FDR) value derived from GSEA analysis is shown. (C) *P < 0.05; **P < 0.01; ***P < 0.001.

Similar loss of BBB integrity in *Tardbp^G348C/+^* knock-ins and in the BrEC-KO mice suggested that similar mechanisms may underlie these effects across cells with either complete or partial loss of nuclear TDP-43. To examine the transcriptional effects of TDP-43 loss, we performed RNA-sequencing analysis of acutely isolated brain ECs from BrEC-KO mice (CD45^neg^, Icam2^+^, CD31^+^, and tomato lectin^+^) and their littermate controls (*n* = 6 versus *n* = 6; tables S1 and S2). We also examined ECs isolated from *Tardbp^G348C/+^* mice and from human primary brain ECs with knockdown of TDP-43 by small interfering RNA (siRNA), with the goal of understanding similarities in gene expression. Because in vitro conditions only poorly replicate in vivo, we examined in vitro responses to altered shear stress and inflammatory stimulus, as well as standard static culture conditions. In general, as with barrier defects, changes in transcript level were more robust in the ECs derived from BrEC-KO mice and siTDP-43–treated human brain ECs than in cells derived from *Tardbp^G348C/+^* mice. Focusing on the changes in BrEC-KO mice, we observed that many transcriptional changes changed in the same direction in *Tardbp^G348C/+^* mice or siTDP-43–treated human brain ECs and often both (Fig. 5, A and B and table S3). Gene set enrichment by Enrichr showed that genes induced by loss of KLF2 and KLF4 or exposure of ECs to disturbed flow were increased. *CXCR4*, a gene increased in FTD, is increased in both BrEC-KO cells in vivo and human brain ECs in vitro (*32*). *ST6GALNAC2*, which drives sialyation of glycans, was reduced and may contribute to the reduced detection of sialyated glycans by tomato lectin (figs. S2 and S13). *KLF2*, a critical transcriptional regulator of endothelial quiescence, is also strongly reduced in mouse brain endothelium in vivo and also in human brain ECs. Analysis of increased transcripts by a gene set enrichment analysis (GSEA) incorporating relative ranking of alterations showed that the genes most induced by loss of TDP-43 were enriched in Hallmark pathways for EF2 targets, G_2_-M checkpoint, and mitotic spindle, while down-regulated genes indicated lower Wnt/β-catenin pathway activity (fig. S16 and table S4). This was interesting to us because we had observed reduced Wnt/β-catenin signaling neurodegenerative capillary ECs with reduced levels of TDP-43 (*23*).

To more carefully examine endothelial specific transcriptional responses in key pathways, we obtained gene set signatures from the literature (Wnt/β-catenin and KLF2/KLF4 responses in mouse and human ECs) and from our own data [gene expression changes in disease-associated endothelium in AD and ALS-FTD (*23*) and endothelial response to disturbed flow (*33*) and tumor necrosis factor–α (TNF-α) stimulation]. The most significantly altered genes were selected in each perturbation (table S5). The focus on endothelial-specific transcriptional responses revealed a strong correlation between transcripts induced in BrEC-KO ECs and the transcripts induced in neurodegenerative diseases AD and ALS-FTD in humans [normalized enrichment score (NES) of 1.6, false discovery rate (FDR) < 0.0001; fig. S16] and also genes suppressed by Wnt/β-catenin agonist CHIR (NES of 1.8, FDR < 0.00001; fig. S16) and genes induced by TNF-α stimulation (NES of 1.6, FDR < 0.00001; fig. S16).

We were interested in which of the identified pathways were consistently affected between the BrEC-KO brain ECs and *Tardbp^G348C/+^* brain ECs and also human brain ECs with TDP-43 suppression. To do this, we performed the same ranked GSEA analysis in the differential gene expression patterns in each of these comparisons. This included the BrEC-KO data generated above and also similar data from *Tardbp^G348C/+^* brain ECs in vivo and both of these cells in vitro and human primary brain ECs in vitro with or without TDP-43 suppression with two different siRNA. In total, there were 80 conditions examined (table S1, RNA_seq_samples) with nine differential comparisons (table S1, ComparisonsDESeq2). By performing this analysis across a wide range of tissues with TDP-43 dysfunction (KO, siRNA, and disease-associated mutation), we hypothesized that key transcriptional responses would be consistently altered across them. By repeating the GSEA analysis for enriched pathways across all of these comparisons, we found consistent regulation of the similar pathways in BrEC-KO cells and human siTDP-43 cells (Fig. 5C). Similarities between datasets highlighted the KLF2/KLF4 pathway and a direct correlation with the transcriptional signatures that we recently defined in capillaries of ALS-FTD and AD donors with reduced nuclear TDP-43 levels (boxes in Fig. 5C).

**Fig. 6.**
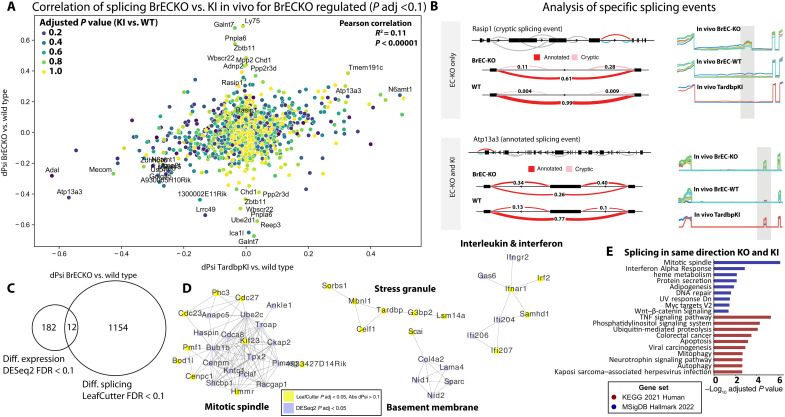
TDP-43 loss results in mis-splicing of key endothelial transcripts. (**A**) Graph shows individual splicing events detected by LeafCutter analysis and significantly different between BrECKO and littermate controls, and correlation with the regulation of the same splicing events in a comparison between *Tardbp^G348C/+^* mice and littermate controls. (**B**) LeafCutter (LeafViz) analysis of example splicing events differentially regulated in BrEC-KO cells in vivo, some of which are also seen in KI cells in vivo. Line plots to the right show read density in each of the replicates across the alternatively spliced region. (**C**) Venn diagram showing correlation between the transcripts affected by differential expression or splicing in the comparison between BrECKO and littermate controls. (**D**) Examples of the top pathways enriched among transcripts affected by either expression or splicing in BrECKO mice, by String analysis in Cytoscape. (**E**) Pathways most affected by significantly regulated splicing changes that were consistently regulated (same direction) in BrECKO and *Tardbp^G348C/+^* mice, relative to their littermate controls. UV, ultraviolet.

The relatively muted transcription level changes in *Tardbp^G348C/+^* ECs was unexpected, given the strong in vivo and in vitro phenotypes in these cells. However, TDP-43 is also known to regulate mRNA splicing. Therefore, we examined RNA splicing across these conditions by LeafCutter, a relatively stringent splicing analysis tool (table S7) (*34*). Filtering on transcripts significantly affected by BrEC-KO TDP-43 deletion in vivo (*P* adjusted < 0.05, *n* = 6 versus *n* = 6), we assessed the overlap with splicing changes in *Tardbp^G348C/+^* mice (Fig. 6A). Consistent with prior observations in whole brain tissues, some splicing events (e.g., *Sort1*) were inversely regulated by deletion versus point mutation (*35*), but the correlation was generally positive [Pearson coefficient of determination (*R*^2^) = 0.11, *P* < 0.0001]. TDP-43 is known to exhibit dose-dependent effects on splicing (*2*); therefore, the splicing events regulated in common may indicate those most sensitive to TDP-43 loss. As inclusion of cryptic exons has been linked to mRNA degradation by NMD, we looked for overlap between alternatively spliced transcripts and those that were changed at the mRNA level. Generally, there was very little overlap, suggesting that, at least for this set of splicing changes, the impact on mRNA levels is limited (Fig. 6C). To understand the pathways affected by both splicing and expression, we performed a joint analysis of transcriptome changes in the BrEC-KO mice affected by both. One of the most prominent changes was on mitotic spindle associated genes, and other notable pathways included stress granules, basement membrane, and interleukin and interferon signaling (Fig. 6D). To more specifically address changes occurring in the endothelium in vivo in both BrEC-KO mice and the *Tardbp^G348C/+^* mice, we examined splicing events going in the same direction in both. Again, we found effects on the mitotic spindle and interferon, TNF, Myc, and Wnt signaling (Fig. 6E), suggesting that, despite little overlap between individual transcripts affected by loss of TDP-43 and the splicing changes that occurred, similar pathways are indicated by either approach.

Thus, an analysis of consistent alternative splicing patterns and gene expression in *Tardbp^G348C/+^* mice reveals transcriptional changes partially overlapping those of BrEC-KO mice and highlights effects on signaling pathways required for BBB maintenance (Klf2/Klf4 flow response, Wnt, and TNF/NF-κB) and cytoskeletal organization (mitotic spindle). Similar transcriptional responses were observed in ECs with reduced levels of TDP-43 in human tissues (*23*), indicating that reduced TDP-43 disrupts these critical pathways in the endothelium in ALS-FTD and AD.

### Endothelial loss of TDP-43 causes FTD-like phenotypes

*Tardbp* knock-in mouse models of ALS-FTD, like *Tardbp^G348C/+^*, may affect multiple cell types within the brain. To determine whether hallmarks of FTD might be linked to TDP-43 loss from the endothelium, we examined these hallmarks 11 months after postnatal excision of TDP-43.

**Fig. 7.**
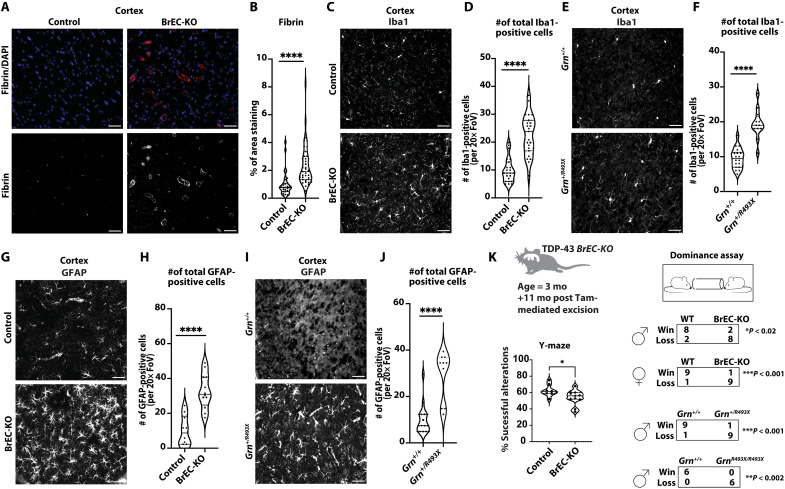
Pathological and behavioral consequences of chronic endothelial TDP-43 loss. (**A**) Representative immunofluorescence images of fibrin deposition in mouse brain frontal cortex sections from 8- to 11-month-old mice (*n* = 3 BrEC-KO and *n* = 3 littermate controls) are shown. (**B**) Quantification of data, with each data point representing the fluorescence image intensity in one image. (**C** and **E**) Iba1 staining of microglia in the mouse brain cortex reveals consistent results across *n* = 3 BrEC-KO and *n* = 3 littermate controls mice (*n* = 3), as well as *n* = 3 *Grn^R493X/+^* and *n* = 3 littermate controls mice (*n* = 3), and (**G** and **I**) GFAP staining of astrocytes reveals a substantial increase in astrocyte numbers, resembling astrogliosis observed in FTD. (**D**, **F**, **H**, and **J**) Quantification of data with each data point representing the number of activated cells in an image, with multiple images per mouse. (**K**) Y-maze and tube dominance test results for BrEC-KO mice. For tube dominance test, *Grn^R493X/+^* mice are included as a positive control, showing a high loss percentage in both models. [(B), (D), (F), (H), and (J)] FoV is 0.16 mm^2^. Data are presented as means ± SEM. Statistical analysis was conducted using an unpaired Mann-Whitney test, with significance levels indicated as follows: ****P < 0.0001. [(A), (C), (E), (G), and (I)] and *P = 0.044 (K). Scale bars, 50 μm.

First, we stained for fibrin, which is deposited near compromised brain vessels and contributes directly to neuronal damage (*27*, *36*, *37*). We found increased fibrin staining around the vessels in the cerebral cortex and midbrain (Fig. 7, A and B, fig. S17, A and B). Fibrin deposition has been linked to microglial activation, a common response observed in cases of AD and ALS-FTD as well as animal models of the diseases (*38*–*40*). Therefore, we stained sections from BrEC-KO and littermate controls with Iba1. We found increased numbers of microglia in BrEC-KO, consistent with increased microglial activation (Fig. 7, C and D; *P* < 0.0001; fig. S18, A and B). Notably, we used another mouse model of FTD, based on a knock-in mutation in progranulin (*38*), *Grn^R493X/+^*, and found that the increase in microglial numbers in the BrEC-KO closely paralleled both this mouse model (Fig. 7, E and F, and fig. S18, C and D) and the *Tardbp^G348C/+^* mice (Fig. 1, I and J). Astrogliosis has also been observed in FTD, where it occurs before neuronal loss. Therefore, we stained and counted GFAP^+^ cells. We found a substantial increase in the number of astrocytes in BrEC-KO mice and *Grn^R493X/+^* mice relative to littermate controls (Fig. 7, G to J, and fig. S18, E to H), resembling our early observation in *Tardbp^G348C/+^* mice (Fig. 1, G and H). As we had observed in *Tardbp^G348C/+^*mice, BBB damage was not associated with an influx of CD45^high^ hematopoietic cells (fig. S5). In addition, despite no effect on neuronal TDP-43, we observed an increase in phospho-Tau in neurons, indicating neuronal damage (fig. S4). Thus, the increased perivascular fibrin deposition, gliosis, and phospho-Tau indicate chronic damage to the brain parenchyma resulting from endothelial loss of TDP-43. As with BBB defects, these effects were generally stronger in BrEC-KO mice than in *Tardbp^G348C/+^* mice.

Parenchymal changes in the brain are reminiscent of changes that occur in neurodegeneration. The Y-maze has been used to demonstrate memory impairments in mice, including both AD models and *Tardbp* ALS-FTD knock-in models. We tested BrEC-KO mice, 12 months after gene excision, and their littermate controls in Y-maze and found a significant reduction in recall of previously visited Y-maze arms (Fig. 7K). Of the behavioral tests most reliably associated with mouse models of FTD, the tube dominance test has accurately segregated FTD models from controls (*41*). Therefore, we examined both BrEC-KO mice and *Grn*^R493X/+^ mice along with their littermate controls in this assay. As expected, in cage-mate matches, the *Grn*^R493X/+^ mice backed away from their wild-type controls, with a nearly 100% “loss” for both *Grn^R493X/+^* or *Grn^R493X/R493X^* mice (Fig. 7K). We observed a notably similar phenotype in the BrEC-KO mice, whether males or females were examined (Fig. 7K). We observed no differences in basal activity in open field test or rotarod assay (fig. S19). Therefore, in addition to pathological markers of cortical damage in FTD (fibrin deposition glial activation and phospho-Tau), the chronic BrEC-KO mice exhibited defects in memory and social interaction.

## DISCUSSION

Here, we show that TDP-43 has a critical function in the maintenance of the BBB. A single mutated allele of *Tardbp*, harboring a point mutation found in patients with ALS-FTD, is sufficient to cause an age-dependent disruption of BBB in vivo. This coincides with reduced cell-cell junctions and barrier properties in isolated primary brain ECs from the mice, indicating a cell-autonomous effect. Modeling the endothelial loss of TDP-43 that occurs in these cells with *Cdh5(PAC)-CreERT2*–mediated deletion resulted in systemic organ dysfunction and lethality. Specific deletion of TDP-43 in the brain endothelium (BrEC-KO) bypassed these systemic effects to isolate the effects of TDP-43 loss in the brain vasculature and demonstrate an acute loss of junctional VE-cadherin and barrier within days, followed by hallmarks of FTD in the cerebral cortex over several months, including microgliosis and astrogliosis, increased phospho-Tau, and behavioral defects. Direct comparison of the transcriptional effects of TDP-43 loss in the murine endothelium with single-nucleus data from TDP-43–deficient ECs of human ALS-FTD and AD tissues shows significant overlap in affected signaling pathways, including the regulation of pathways essential for endothelial barrier function, including Wnt, NF-κB, flow-mediated responses, and the cytoskeleton. Together, these data demonstrate that even a partial disruption of TDP-43 in the endothelium can lead to large effects on the cytoskeleton and junctional complexes and that the specific deletion of TDP-43 from the brain endothelium models key aspects of endothelial dysfunction in ALS-FTD and AD.

### TDP-43 and BBB maintenance

Despite the early focus on TDP-43 function in neurons as a mediator of ALS-FTD, it is now appreciated that this fairly ubiquitous splice factor is likely to affect the function of most cells, including fibroblasts, astrocytes, and even cells of the pancreatic islet (*6*–*10*, *42*). It has also become clear that the transcriptional effects of TDP-43 dysfunction exert cell and species-specific effects on RNA splicing. Most notably, cryptic exons, which appear upon loss of TDP-43, show little overlap between stem cells, neurons, and muscle (*43*). Penetrance of TDP-43–associated ALS-FTD is both incomplete and highly variable in onset, with similar causal *TARDBP* mutations linked to a wide range of phenotypic presentations in different individuals (*11*, *12*). One possibility is that, despite the expression of the mutant protein in multiple cell types, that phenotypic presentation is determined by cell-type–specific vulnerability.

Here, we reveal a direct effect on the BBB in animal models that occurs, in part, through cytoskeletal disruption and a loss of cell-cell junction proteins. Our data are consistent with prior work showing that the loss of TDP-43 orthologs in zebrafish led to specific vascular defects (*44*, *45*). Although this work did not examine endothelial or BBB function, it did identify defects in matrix interaction, notably increased endothelial expression of FN1 and Integrin α4. Recent work in mouse models, using the same *Cdh5(PAC)-CreERT2* approach used here, confirmed these angiogenesis defects and also showed impaired matrix interactions (*29*). Although these mice exhibited reduced junctional protein expression, microgliosis, hemorrhage in the central nervous system, and lethality with 2 to 3 weeks of gene excision, dissecting primary and secondary effects in the context of severe multi-organ dysfunction and lethality is challenging. Nevertheless, as AAV–TDP-43 treatment of cultured ECs also affects junctional proteins in vitro (*46*, *47*), several lines of evidence now point to defects in matrix adhesion, cytoskeletal dysfunction, and impaired cell-cell junction complex resulting from endothelial TDP-43 dysfunction. Our data do not exclude effects on receptor mediated or general transcellular transport mechanisms, which may also be involved in BBB dysfunction. Regardless of the mechanism, the data clearly demonstrate the sensitivity of the brain endothelium to TDP-43 dysfunction and support that the reduced levels of TDP-43 that we have recently observed in ECs in ALS, FTD, and AD capillaries are connected to barrier dysfunction (*23*). A critical question now is: What causes the observed age-dependent TDP-43–linked dysfunction? If a similar loss of nuclear TDP-43 occurs in the endothelium in sporadic cases of ALS-FTD and AD (*23*), then what are the molecular drivers?

### Hallmarks of FTD with loss of TDP-43 and BBB

FTD is defined by a loss of neuron function in the frontal temporal lobe, broadly diminished social conduct and foresight, and also reduced language and speech. Rodent models of FTD are imperfect representations of these behaviors, but the most consistent behavioral phenotypes in mouse models of FTD (e.g., *Grn^+/−^*, *Grn^R493X/+^*, or *Tardbp^Q331K/+^*) are reduced marble burying (considered a measure of perseverative behavior) (*35*), reduced Y-maze/novel object exploration (a measure of memory) (*35*), retreat from open spaces (anxiety) (*48*, *49*), and social dominance over littermate control mice (social interaction) (*41*). We identify defects in both memory and social interaction in mice with long-term loss of brain endothelial TDP-43 and validate the previously observed retreat of Grn heterozygous loss-of-function mice in social interactions. We did not observe clear motor neuron defects, which could indicate either that other cell-specific mutations, for example, in the motor neurons themselves, are required for that disease manifestation. However, that the loss of TDP-43 from brain ECs alone is sufficient to drive key aspects of FTD is remarkable.

In BrEC-KO mice, behavioral defects are likely a consequence of chronic leak of multiple plasma components and fibrinogen in particular, across the BBB, leading to activation of microglia and astrocytes and damage to neurons (*19*). Acute vascular leak has been linked to cognitive defects, for example, following postnatal deletion of the S1P receptor in the endothelium (*50*) and after acute astrocyte or pericyte deletion (*26*, *51*). Each of these perturbations leads to different relative levels of BBB dysfunction. For example, pericyte depletion results in substantial changes in blood flow and vascular density within just days of pericyte depletion, coinciding with edema, leak of large molecules (immunoglobulin G), and subsequent loss in neurons and impaired novel object recognition (*51*). In contrast, partial astrocyte depletion led to reversible BBB leak to the small-molecule cadaverine (~175 Da) and similarly reversible effects on astrogliosis and microgliosis, coinciding with resolution of behavioral defects within 2 weeks (*26*). An even milder effect on the BBB is observed with S1P receptor deletion in adult mice, which showed no astrogliosis or cognitive dysfunction despite leak of low–molecular weight dyes (~175-Da cadaverine and 3-kDa dextran) (*50*). The consequences of BBB dysfunction caused by endothelial by loss TDP-43 is relatively severe but is likely to be exacerbated by additional stressors. In future work, it will be important to understand additive effects caused by endothelial loss of TDP-43 and other stressors of neuronal function.

### Cytoskeletal alterations as a basis of BBB defects

Our data reveal specific pathways associated with microtubules and extracellular matrix–integrin interactions that were altered across a range of models, including *Tardbp* deletion in vivo and in vitro, siRNA in human cells in vitro, and FTD-associated mutations linked to reduced nuclear levels of TDP-43. Nuclear levels of TDP-43 can affect RNA splicing in a dosage-sensitive manner in neurons (*2*) and often in a species-dependent manner (*4*). Low levels of TDP-43 depletion affect sensitive targets, and complete depletion of TDP-43 affects even the most resistant targets. In our data, FTD mutations in *Tardbp* result in only a partial loss of nuclear function. Nevertheless, we see similar pathways affected by both partial and complete loss of *Tardbp*, for example, centrosome, DNA replication, proteasome, and TNF/NF-κB signaling. The DNA replication defects are consistent with DNA replication defects in cells, resulting, in part, from the development of R-loops during replication (*52*). Recent data support an association between TDP-43 and the centrosome, although its function there is not yet clear (*53*). There is also literature to support an interaction between NF-κB pathway transcription factor RELA/p65 and TDP-43; however, at least in microglia, the interaction leads to downstream NF-κB target gene stimulation, opposite to what we see in the endothelium (*54*). That partial and complete loss of TDP-43 leads to similar pathway effects suggests that these pathways in the endothelium are highly sensitive to alterations in TDP-43 function.

In summary, our data demonstrate that ECs are highly sensitive to the levels of TDP-43. As nuclear levels are reduced by ALS-FTD–associated mutations and reduced endothelial TDP-43 is also observed in sporadic ALS-FTD and AD, we propose that reduced endothelial TDP-43 contributes to BBB permeability and cognitive dysfunction.

## MATERIALS AND METHODS

### Mice

For the pan-endothelial deletion of *Tardbp*, *Cdh5(PAC)-CreERT2* and *Tardbp^lox/lox^* mice were used as previously described (*55*, *56*). They were intercrossed to create the EC-KO mice [*Cdh5(PAC)-CreERT2; Tardbp^lox/lox^*] and littermate controls (*Tardbp^lox/lox^*) used in this study for brain endothelium–specific deletion of Tardbp, *Slco1c1-CreERT2*, and *Tardbp^lox/lox^* mice were used; the Slco1c1-CreERT2 line has been previously described (*30*). These mice were intercrossed to create the BrEC-KO mice (*Slco1c1-CreERT2; Tardbp^lox/lox^*) and littermate controls (*Tardbp^lox/lox^*). Grn^R493X/R493X^ mice, previously published (*38*), were backcrossed to C57BL/6J mice and then intercrossed to generate littermates *Grn*^*R493X/R493X*^, *Grn*^*R493X/+*^, and *Grn*^*+/+*^ controls. EC-KO mice are on a C57BL/6 background, BrEC-KO mice are on a mixed background of C57BL/6 and FVB/NJ, and GrnKI mice are on a C57BL/6 background. Mice were used in paired groups of males and females. When delivered, tamoxifen (Sigma-Aldrich) was given intraperitoneally (three 1-mg doses) to all mice in experimental cohorts, dissolved in sunflower oil at 10 mg/ml.

For the generation of *Tardbp^G348C/+^* mice, C57BL/6J single-cell zygotes were microinjected with CRISPR-Cas9 guide RNAs flanking the Ala^315^ to Gly^348^ positioned in exon 6 of the canonical *Tardbp* gene and a synthesized 200-nucleotide donor double-stranded DNA containing the following mutations: A315T (GCT->ACT), M337V (ATG->GTG), and G348C (GGC->TGC). The objective of the targeting experiment was to determine whether a founder mouse containing all three clinical ALS-associated mutations in *Tardbp* could be recovered. This was not successful. However, founders were recovered that contained the G348C allele. Progeny derived from these founders was wild type at both A315T and M337, indicating that the donor DNA fragmented and only the G348C variant allele was established in the genome. A line was established from founder 186 and designated as JAX strain no. 028435 and the G348C allele designated as *Tardbp^em9Lutzy^*. For our purposes in this manuscript, we refer to this line as *Tardbp^G348C^*, which refers to the amino acid change in TDP-43 protein. All mice were housed and handled in accordance with protocols approved by the University of Connecticut Health Center for Comparative Medicine and the Institutional Animal Care and Use Committee.

#### Measurement of vascular leak

Mice received a retro-orbital injection of tomato lectin 488 (50 μl; DL-1174-1, Vector BioLabs), 3-kDa Texas Red–dextran [50 μl; D3329, Thermo Fisher Scientific; 10 mg/ml in phosphate-buffered saline (PBS)], EZ-Link Sulfo-NHS-Biotin (100 μl; A39256, Thermo Fisher Scientific; 1 mg/0.4 ml in PBS). After 15 min of circulation, mice were euthanized by CO_2_, and heparinized plasma samples were collected via cardiac puncture.

##### Direct dye measurement in the whole brain

After removing the meninges, the forward half-hemisphere was used for dye extraction in 0.1% Triton X-100 in 1× PBS. Following bead homogenization, the soluble supernatant containing Texas Red–dextran was measured on a fluorescent plate reader, and the signal was then normalized to brain weight and plasma concentration of dye.

##### Analysis of dye and NHS-biotin leak in brain section

The rear half-hemisphere was fixed in 4% paraformaldehyde (PFA), dehydrated in 30% sucrose, and embedded in OCT on LN_2_-cooled metal block for sectioning at 10 and 50 μm. Samples were directly imaged for 3-kDa Texas Red–dextran and stained with streptavidin for imaging of NHS-biotin.

#### Direct labeling and visualization of blood vessels with lipophilic carbocyanine dye DiI

The DiI labeling experiment was performed according to the published protocol (*57*). Mice were euthanized with CO_2_, and the heart was exposed. A needle was inserted into the left ventricle, and the right atrium was snipped. The animal was perfused with 10 ml of PBS at 1 ml/min, followed by 10 ml of 4% PFA for tissue fixation. After fixation, 10 ml of DiI working solution was perfused to label the vasculature. Immediately following DiI perfusion, the brain was perfused with 10 ml of PBS to remove unbound DiI. The brain was then dissected, sectioned, and rinsed twice with 500 μl of 1× PBS. Sections were mounted on glass slides with Fluoromount, covered with coverslips, and visualized using a fluorescence microscope with a red filter set (550-nm excitation and 567-nm emission).

#### Isolation of brain ECs for direct analysis of RNA

Samples with dye injection were prepared as described above. After removing the meninges, one brain hemisphere was minced and digested in Hanks’ balanced salt solution (HBSS) and density filtered over 22% Percoll as previously described (*58*). The resulting pellet was resuspended in 300 μl of HBSS/bovine serum albumin (BSA)/glucose buffer along with the following antibodies: allophycocyanin-cyanine7 (Cy7) CD45, phycoerythrin (PE) CD31, PE-Cy7 ICAM1, Alexa Fluor 647 ICAM2 at 1:100, and Live/Dead at 1:1000 (L34962, Invitrogen), for sorting of CD45^neg^, lectin^+^, ICAM2^+^, and CD31^+^ ECs, respectively (BD FACSAria III Cell Sorter, BD Biosciences).

#### Isolation of murine brain ECs for culture and in vitro treatment

Microvessels were isolated from brain tissue after removing the meninges, as described (*59*), except that ECs were allowed to grow out from microvessels on collagen I–coated plates after digestion and myelin depletion. Cells were maintained in vascular cell basal medium [American Type Culture Collection (ATCC), PCS-100-030] with vascular endothelial growth factor (VEGF) additive kit Endothelial Cell Growth Kit–VEGF (ATCC, PCS-100-041), supplemented with Primocin (InvivoGen, 100 μg/ml), a broad-spectrum antibiotic. Cells were cultured at 3% O_2_ and 5% CO_2_, with N_2_ balancing the gas mixture. After 1 day in culture, puromycin (InvivoGen) was added to the medium at a concentration of 1 μg/ml. After 2 days, puromycin medium was removed. The cells were then sorted using ICAM2 (rat anti-mouse CD102; clone 3C4; catalog no. 105602, BioLegend) and purified (Dynabeads Protein G 10003D). Purity of the isolated cells was assessed by immunostaining for pan-endothelial marker VE-cadherin and brain vascular tight junction markers Cldn5 and ZO-1.

#### Human primary brain ECs and in vitro treatment

De-identified primary human brain ECs were obtained from a commercial source (Cell Biologics). Cells were maintained in vascular cell basal medium (ATCC, PCS-100-030) with VEGF additive kit Endothelial Cell Growth Kit–VEGF (ATCC PCS-100-041). Endothelial identity and expression of brain vasculature tight junction markers were confirmed by staining for VE-cadherin, Pecam1, Cldn5, and ZO-1.

#### Dextran transwell assay

Murine brain ECs were plated on a collagen-coated transwell membrane (Costar 3401) at confluency in ATCC and VEGF medium. Medium in the upper well was replaced with 10-kDa FITC-dextran (0.1 mg/ml; D1820, Invitrogen) in culture medium and sampled at the indicated time points from the bottom transwell for analysis [CLARIOstar Plate Reader (BMG Labtech) at a 494/518-nm wavelength]. Transwell filters were collected at endpoint for analysis of cell coverage by DAPI and phalloidin.

#### Immunofluorescent analysis of brain tissue and cells in culture

Brain samples, with and without dye injection described above, were fixed in 4% PFA and prepared in OCT as described above, before cutting at 10 or 50 μm followed by OCT removal and staining. Murine brain ECs were plated on collagen-coated eight-well chamber slides (C86024, MilliporeSigma). At ~90% confluence, they were washed and fixed with 4% PFA. For staining of both tissue sections and cultured cells, slides were blocked/permeabilized for 15 min in 2% BSA and 0.1% Triton X-100, before staining in a 1:10 dilution of block:PBS and the indicated antibodies overnight at 4°C (see Table 1). After PBS washes, secondary antibodies (1:1000) and DAPI (1:10,000; D9542-1, Sigma-Aldrich) were incubated for 2 hours at room temperature, before washing with PBS and mounting (Fluoromount; F4680, Sigma-Aldrich). To visualize the actin cytoskeleton, the cells were incubated in 5 μl of phalloidin-FITC (5782, Bio-Techne) methanolic stock solution diluted in 200 μl of blocking solution for 1 hour in the dark at room temperature. Images for control and mutant conditions were collected in the same imaging session and with the same settings, using either a Zeiss Axioskop or Zeiss LSM-800 confocal. Raw CZI data (Zeiss data format) were obtained for analysis in ImageJ/Fiji (National Institutes of Health, Bethesda, MD, USA). Multiple fields from tissues or cells from multiple donors (>3) of each genotype were collected, and data were processed in parallel.

**Table 1.** Antibodies for immunofluorescence microscopy.

Antibody	Catalog number	Provider	Dilution	Antigen retrieval
Anti–TDP-43	#ab109535	Abcam	1:50	Yes
Anti-ERG	# ab196149	Abcam	1:100	Yes
Anti-fibronectin^*^		Richard O. Hynes lab	1:200	No
Anti–collagen IV	#PA1-28534	Thermo Fisher Scientific	1:100	No
Anti-fibrinogen	#A0080	Agilent Dako	1:50	Yes
Anti-GFAP	#13-0300	Thermo Fisher Scientific	1:200	Yes
Anti-Iba1	#ab178846	Abcam	1:100	No
Anti–VE-cadherin	#550548	BD Biosciences	1:100	No
Anti–ZO-1	#ab221547	Abcam	1:50	No
Anti–claudin-5	#ab131259	Abcam	1:50	No
Anti–α smooth muscle actin	#ab5694	Abcam	1:1000	No

*Polyclonal antibodies against fibronectin (297.1) were provided as a gift from Richard O. Hynes lab (Koch Institute for Integrative Cancer Research at Massachusetts Institute of Technology). Antigen retrieval is with heat and 10 mM citrate buffer (pH 6).

#### Nuclei isolation and flow cytometry protocol

The protocol for nuclei isolation and flow cytometry was adapted from our previously published methods (*23*, *60*). In brief, mouse brain tissues (200 mg) were lysed using Nuclei EZ lysis buffer (Sigma-Aldrich, Nuc101), supplemented with ribonuclease inhibitor (0.04 U/μl). The tissues were homogenized using a bullet blender. Nuclei were isolated by centrifugation at 700*g* and subsequently washed with the lysis buffer. The isolated nuclei were stained with the following antibodies: anti–Erg 647, anti–NeuN Cy3, anti–TDP-43 488, and DAPI (see Table 2). The nuclei were then analyzed using a flow cytometer.

**Table 2.** Antibodies for flow cytometry. APC, allophycocyanin; PE, phycoerythrin.

Antibody	Catalog number	Provider	Dilution
Rat PE anti-mouse CD31	#102508	BioLegend	1:100
Rat APC/cyanine7 anti-mouse CD45	#103116	BioLegend	1:100
Rat PE/cyanine7 anti-mouse CD54	#116122	BioLegend	1:100
Rat Alexa Fluor 647 anti-mouse CD102	#105612	BioLegend	1:100
Rabbit Alexa Fluor 488 anti–TDP-43	#ab193842	Abcam	1:200
Mouse anti–NeuN Cy3	#MAB377	MilliporeSigma	1.200

#### Immunofluorescent analysis of whole mount brain tissue

Mice received a retro-orbital injection of tomato lectin 488 (50 μl; DL-1174-1, Vector BioLabs); 15 min postinjection, mice were perfused with 1% PFA, the brain was harvested, and a section of the cortex was excised for subsequent staining. Briefly, sections were blocked/permeabilized for 5 hours in 2% BSA and 0.1% Triton X-100 at room temperature before staining in a 1:10 dilution of block:PBS and the indicated antibodies overnight at 4°C (see Table 1). After PBS washes and a 0.2% BSA and 0.01% Triton X-100 wash, secondary antibodies (1:1000) and DAPI (D9542-1; Sigma-Aldrich, 1:10,000) were incubated for 5 hours at room temperature before washing with PBS and mounting (Fluoromount; F4680, Sigma-Aldrich).

#### RNA isolation and analysis

RNA was isolated from sorted or cultured cells by RNeasy mini or micro columns (QIAGEN), with on-column deoxyribonuclease treatment. RNA was checked for integrity and yield before library preparation (SMART-Seq mRNA LP). Targeted sequencing was 100 million 150 + 150 paired-end reads on a NovaSeq and generally yielded more than 100 million paired-end reads per sample. A full list of these datasets is found in table S1, and data are deposited to Sequence Read Archive (SRA-PRJNA1054818).

Human and mouse FASTQ were aligned (star/2.7.1a) to hg38 or mm10. For gene expression analysis, the following flags were used: --outFilterMultimapNmax 20 –alignSJoverhangMin 8 –alignSJDBoverhangMin 1 –outFilterMismatchNmax 999 –alignIntronMin 10 –alignIntronMax 1000000 –alignMatesGapMax 1000000 –peOverlapNbasesMin 5. Results were assessed by Qualimap. Transcript counts (rest/1.3.0) were performed using the following parameters: –paired-end –calc-ci. Output is in table S2.

Differential gene expression was performed by DESeq2 using RSEM-generated gene counts after STAR alignment as described (*61*). Output is in table S3. For APAlyzer analysis, we followed the APAlyzer protocol from bam files generated by STAR (*62*). Output for primary human brain ECs with and without siTARDBP is in table S4. For splicing analysis by LeafCutter, FASTQ were remapped according to LeafCutter suggested parameters: --twopassMode Basic –alignIntronMin 10 –outSAMstrandField intronMotif. Output was assessed by Qualimap, before analyzing splicing according to LeafCutter protocol (*34*). Data (with mouse genes lifted over to human homolog with BioMart) are contained in table S5.

#### GSEA of similarities in gene expression patterns

To examine overlap in gene sets by expression, we used GSEApy. Briefly, we generated pre-ranked lists from our data on experimental deletion or silencing of TDP-43 in human or mouse brain ECs or from cells of mice with ALS-FTD–associated mutations in *Tardbp* or *Grn*. These ranked lists were filtered for BaseMean expression > 5 to exclude genes that were not expressed at a sufficient level to be called as differential and ranked on the basis of on log_2_ fold change. We then examined the relative enrichment of MsigDB Hallmark (2020) and Kyoto Encyclopedia of Genes and Genomes (2021) pathway gene sets within the ranked list, using GSEApy parameters “log_2_ ratio of classes” and “weighted score type 1” with 1000 permutations. These gene sets were mapped to the mouse homolog using BioMart. In addition, we created custom gene sets from our own data or from publicly available data. The specific datasets used and their parameters are listed in table S6. From these datasets, we extracted the most significantly up-regulated or down-regulated genes, resulting in the generation of two gene sets from each perturbation. These custom gene sets were mapped to the mouse homolog using BioMart if derived from human data and then used in a GSEA search as previously described. NES and adjusted *P* value resulting from these GSEA searches are shown on the associated heatmap. To determine similar pathways, the overlap in pathways is shown in NetworkX, where the overlap between any two datasets is given as the total number of genes in common between the datasets divided by the total number of genes in both datasets (% overlap). Key enriched pathways were plotted in GSEApy to show the placement of pathway genes among the up- or down-regulated transcripts in the datasets. Complete code can be found at https://github.com/pamurphyUCONN/2025_Cheemala (https://zenodo.org/records/14624581), with source files for the Jupyter Notebook available (https://zenodo.org/records/14624526).

#### Statistical analysis

Data include samples from male and female mice. Data were merged if similar responses were observed in both sexes. All data collected from immunofluorescence were analyzed using the unpaired two-tailed Mann-Whitney test unless otherwise specified. All statistical analyses were conducted using GraphPad Prism software (GraphPad Software, La Jolla, California, USA).

#### Behavioral testing

##### Open-field maze test

Mice were tested on the Photobeam Activity System (San Diego Instruments). This system consists of a 16 inch (40.64 cm)–by–16 inch (40.64 cm) clear acrylic box. Mice did not receive any training. Following a 20-min acclimation period, mice were allowed to freely explore for 10 min. The apparatus was cleaned before and between trials using 70% ethanol.

##### Rotarod test

Mice were tested on a rotarod apparatus (ENV-574 M from Med Associates Inc.). Before the trials, mice were given one training session lasting 1 min at 6 rpm. This was followed by three trials in which the rod’s speed was ramped up from 6 to 60 rpm. Mice were brought into the testing room and allowed to acclimate for 20 min before the training session. Once all the mice were on the rotating rod and facing in the correct direction, the training session or trial (and subsequent ramping up of speed) was initiated. Mice were considered “out” either when they fell and broke the beam at the bottom of the apparatus or when the mouse made a full revolution of the rod while hanging on, at which point the beam was tripped manually. The apparatus was cleaned before and between trials using 70% ethanol.

##### Tube dominance test

Mice were brought into the testing room and allowed to acclimate for 20 min before the trials. The tube used was the Tube Dominance Test (Panlab LE899M), a clear polymethyl methacrylate tube measuring 30 cm in length and 3.4 cm in diameter. This tube featured two clear gates, each located 13 cm from one end. Two mice of the same sex from the same cage but of different genotypes were placed at opposite ends of the tube and allowed to enter. Once both mice were entirely inside the tube with their noses at the gates, the gates were removed, and a timer was started. A mouse was marked as the loser when both of its feet were out of the tube, and a time was recorded. Trials that lasted more than 2 min were repeated at the session’s end. Trials in which a mouse turned around or passed its opponent were abandoned. While the mice were not specifically trained for the tube, the same mice were used for multiple trials on the same day. Subsequent trials alternated the starting positions of the mice. Between and before trials, the tube was sprayed with ethanol and dried with a paper towel.

##### Y-maze

Mice were brought into the testing room and allowed to acclimate to the space for 30 min before the assessment. Mice were placed in one arm of the Y-maze and allowed to freely explore for 5 min. Movement in the apparatus was recorded using the ANY-maze tracking software (Stoelting, Chicago, IL). The innate tendency of mice to spontaneously alternate between the three arms and enter the least recently visited arm was assessed by determining the percentage of successful alternations [(number of correct alternations/(total arm entries − 2) * 100]. The apparatus was cleaned before and between trials using 70% ethanol.
