# Supplementary material for: Amyotrophic lateral sclerosis and frontotemporal dementia mutation reduces endothelial TDP-43 and causes blood-brain barrier defects
Source: Sci Adv. 2025 Apr 16;11(16):eads0505. doi: 10.1126/sciadv.ads0505 (PMC12002129; doi:10.1126/sciadv.ads0505)
Supplement: Supplementary file 1 — Figs. S1 to S20 Legends for tables S1 to S7 [file sciadv.ads0505_sm.pdf]

Science Advances
AAAS

Supplementary Materials for

Amyotrophic lateral sclerosis and frontotemporal dementia mutation reduces endothelial TDP-43 and causes blood-brain barrier defects

Ashok Cheemala *et al.*

Corresponding author: Patrick A. Murphy, pamurphy@uchc.edu

Sci. Adv. **11**, eads0505 (2025)
DOI: 10.1126/sciadv.ads0505

The PDF file includes:

Figs. S1 to S20
Legends for tables S1 to S7

Other Supplementary Material for this manuscript includes the following:

Tables S1 to S7

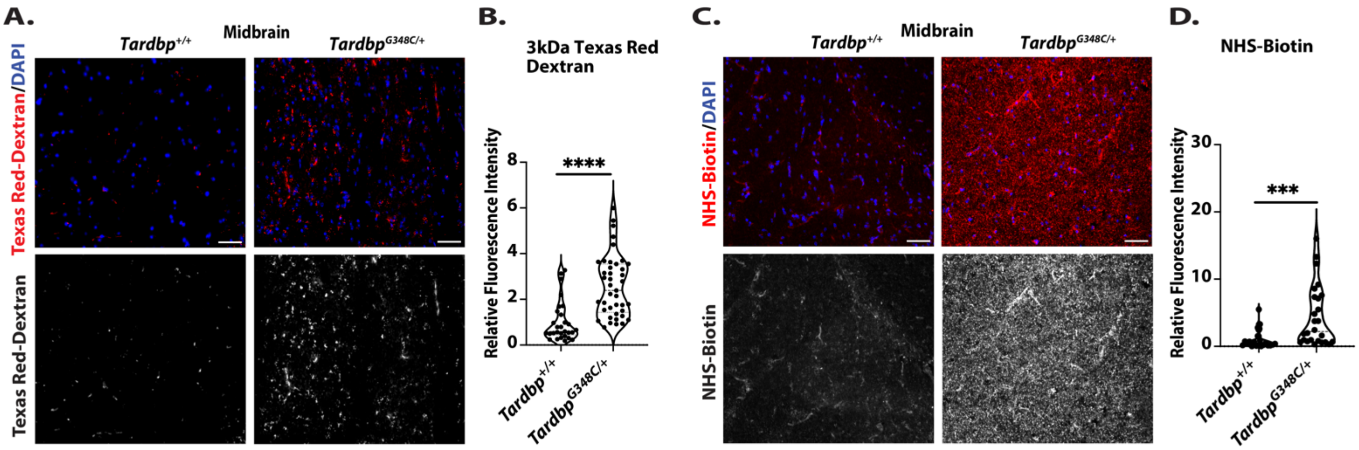

SI Figure 1. Assessment of Blood-Brain Barrier Permeability. (A) The representative immunofluorescence images of 3kDa Texas Red-dextran leakage in mouse midbrain sections from 10-11-month-old mice (n=3 *Tardbp*$^{+/+}$ and n=3 *Tardbp*$^{G348C/+)}$ are shown. (C) NHS-Biotin. (B, D) Quantification of data, with each data point representing the fluorescence image intensity in one image, with multiple images per mouse. Scale bars, 50 µm. Data are presented as means ± SEM. Statistical analysis was conducted using an unpaired Mann Whitney test, with significance levels indicated as follows: ***P 0.0001, ****P<0.0001.

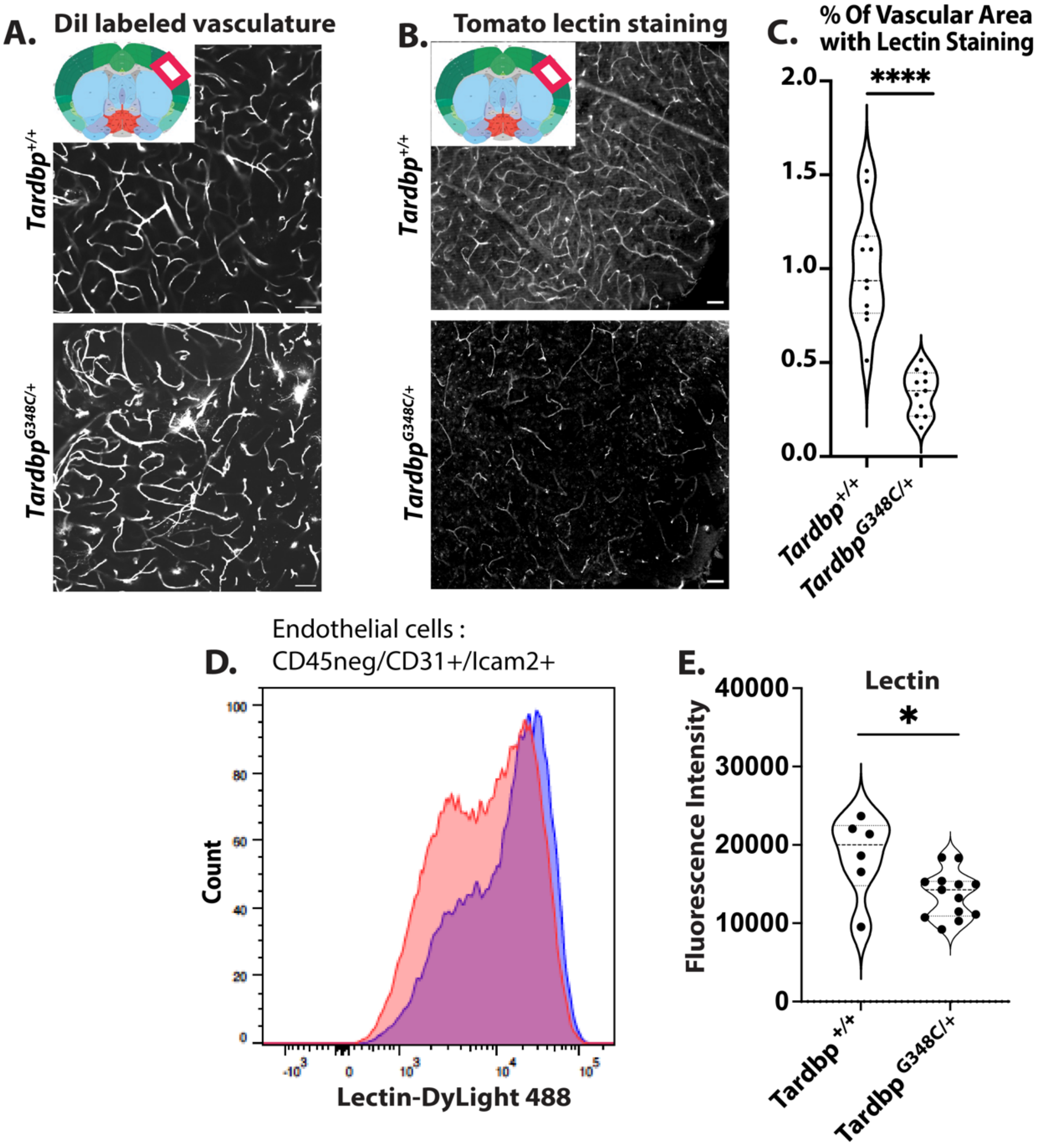

SI Figure 2. Tomato lectin staining of endothelium in *Tardbp$^{G348C/+}$* mice. (A&B) Representative images of (A) DiI perfusion labeling and (B) tomato-lectin staining of the cortical vasculature in *Tardbp$^{+/+}$* and *Tardbp$^{G348C/+}$* mice. (C) Quantification of data, with each data point representing the fluorescence image intensity in one image. *Tardbp$^{G348C/+}$* mice and littermate controls (N=3 and N=3). (D) Flow cytometry plot showing lectin+ staining intensity of sorted endothelial cells from *Tardbp$^{G348C/+}$* mice and littermate controls, and (E) quantitation of mean lectin staining in sorted endothelial cells (N=13 and N=6). Scale bars, 50 µm. Data are presented as means ± SEM. Statistical analysis was conducted using an unpaired Mann Whitney test, with significance levels indicated as follows: *P<0.05, ****P<0.0001.

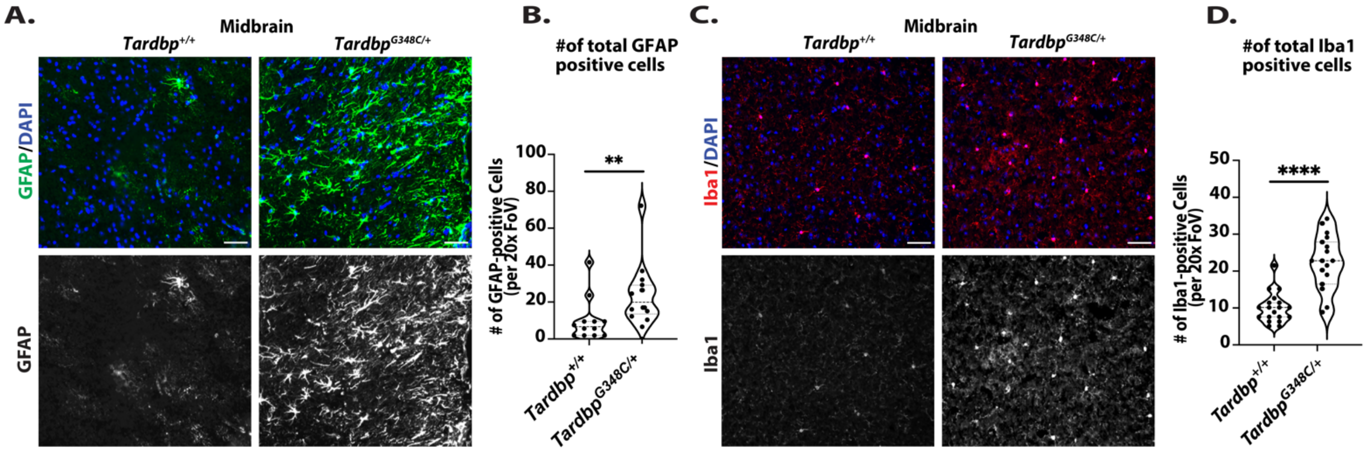

SI Figure 3. Assessment of Astrocyte (GFAP) and Microglia (Iba1) Activation. (A) The representative immunofluorescence images of GFAP staining of astrocytes, and (C) Iba1 staining of microglia in the mouse midbrain reveal consistent results across *Tardbp*$^{+/+}$ mice (n=3) and *Tardbp*$^{G348C/+}$ mice (n=3). (B, D) Field of view (FoV) is 0.16mm^2. Quantification of data with each data point representing the number of activated cells in an image, with multiple images per mouse. Scale bars, 50 µm. Data are presented as means ± SEM. Statistical analysis was conducted using an unpaired Mann Whitney test, with significance levels indicated as follows: **P 0.0023, ****P<0.0001.

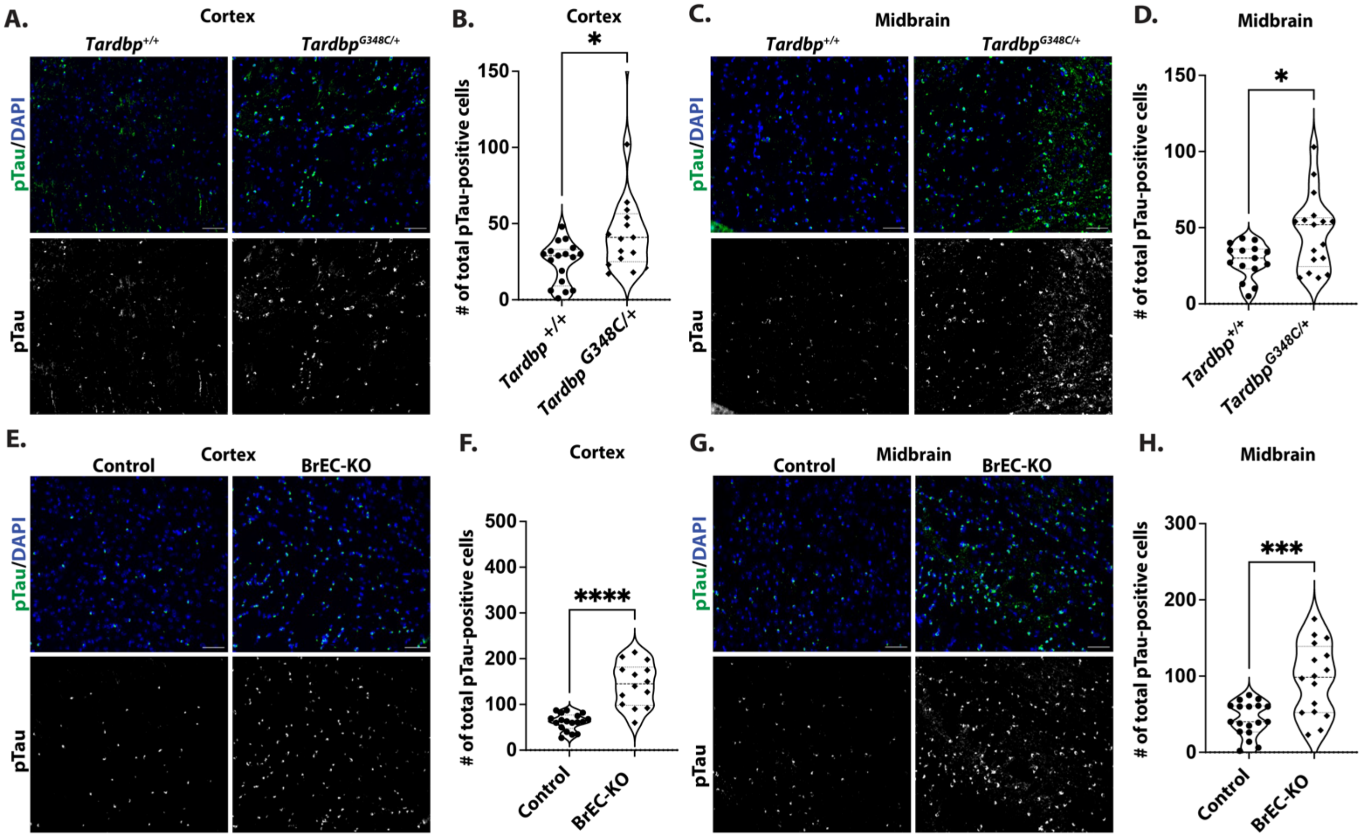

SI Figure 4. Increased phospho-Tau staining in neurons of *Tardbp*$^{G348C/+}$ and BrEC-KO mice.

(A, C) Representative immunofluorescence images showing increased phospho-Tau staining in neurons of the frontal cortex (A) and midbrain (C) sections from 10–11-month-old wild-type *Tardbp*$^{+/+}$ mice (n=3) and their heterozygous littermates, *Tardbp*$^{G348C/+}$ mice (n=3). (E, G) Similar increases in phospho-Tau staining are observed in the cortex (E) and midbrain (G) of 8–11-month-old BrEC-KO mice (n=3) compared to their littermate controls (n=3). (B, D, F, H) Field of view (FoV) is 0.16mm^2. Quantification of phospho-Tau-positive neurons, with each data point representing the number of phospho-Tau-positive neurons per image. Multiple images were analyzed per mouse. In both *Tardbp*$^{G348C/+}$ mice and BrEC-KO mice, phospho-Tau staining was significantly increased compared to controls. Data are presented as means ± SEM. Statistical analysis was conducted using an unpaired Mann Whitney test, with significance levels as follows: *P < 0.05, ***P<0.001, ****P < 0.0001. (A, C, E, G) Scale bars, 50 µm.

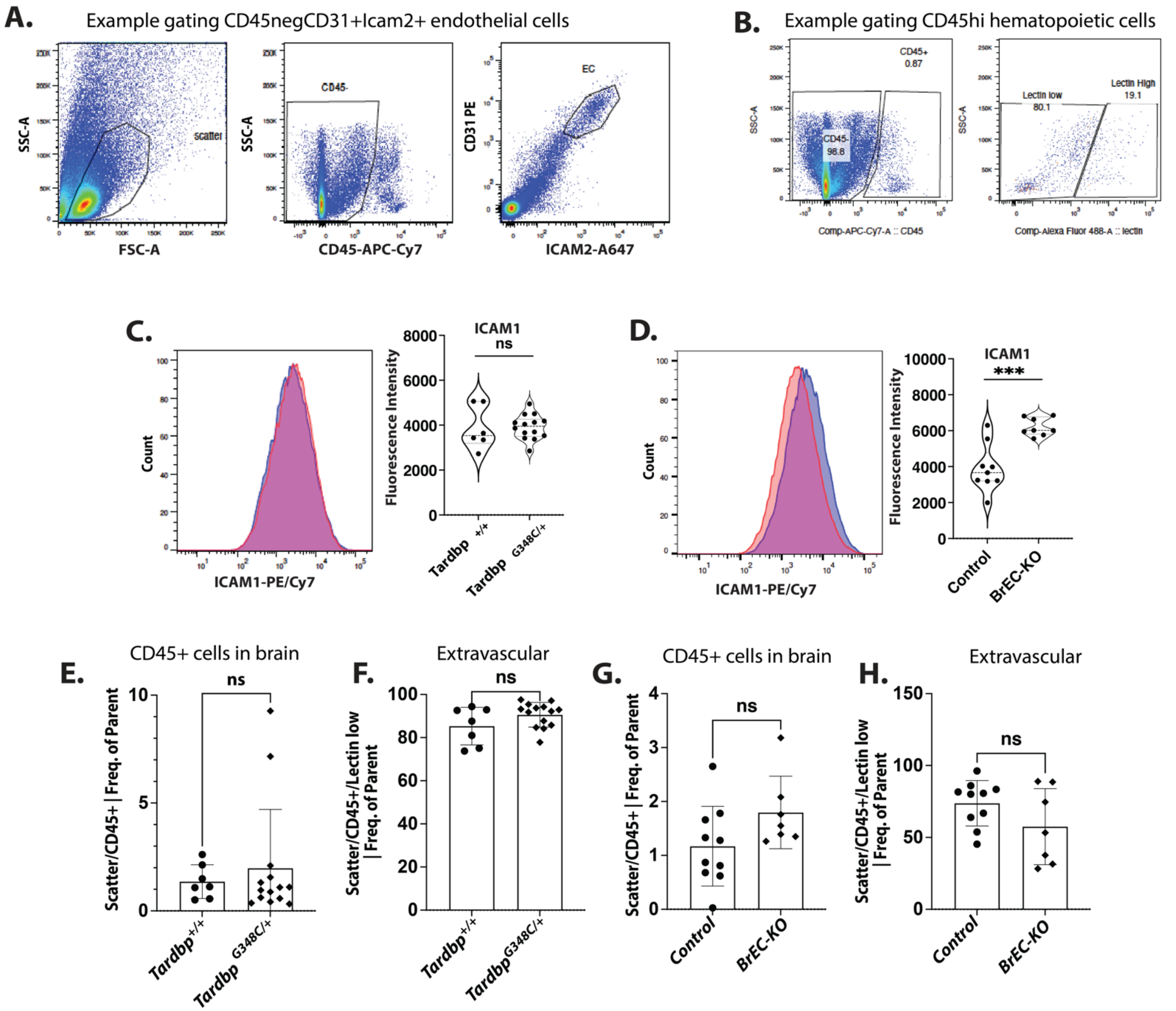

SI Figure 5. Analysis of brain endothelial ICAM1 expression and CD45high hematopoietic cells.
(A) Representative flow cytometry gating strategy for identifying endothelial cells (CD45-negative, CD31-positive, ICAM2-positive). (B) Representative flow cytometry gating strategy for identifying CD45 high hematopoietic cells, followed by further gating to distinguish lectin high and lectin low populations. (C, D) Representative flow cytometry data plots and sample means for ICAM1 expression levels in endothelial cells from *Tardbp*$^{+/+}$ and *Tardbp*$^{G348C/+}$ mice (C) and control and BrEC-KO mice (D). (E, F) Quantification of total CD45+ cells in the brain (E) and extravascular CD45+ cells (F) in *Tardbp*$^{+/+}$ and *Tardbp*$^{G348C/+}$ mice. (G, H) Quantification of total CD45+ cells in the brain (G) and extravascular CD45+ cells (H) in control and BrEC-KO mice. Data are presented as means ± SEM. Statistical analysis was conducted using an unpaired Mann Whitney test, with significance levels as follows: ns (not significant),***P<0.001.

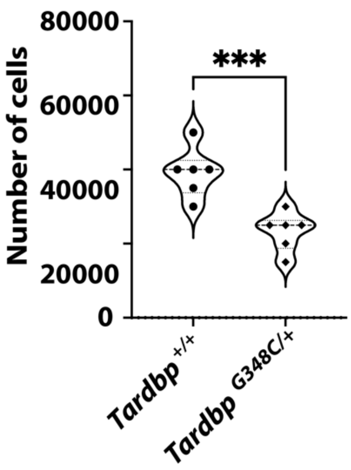

SI Figure 6. Impaired expansion of brain endothelial cells from *Tardbp*$^{G348C/+}$ mice.
Brain endothelial cells (ECs) were purified from *Tardbp*$^{G348C/+}$ mice and their littermate controls. Equal numbers of purified brain ECs (15,000 cells per well in a 12-well plate) were seeded and cultured under identical conditions. Quantification of cell numbers at 48 hours post-plating using hemocytometry, showed significantly reduced proliferation of *Tardbp*$^{G348C/+}$ cells compared to controls. Data are presented as means ± SEM. Statistical analysis was performed using an unpaired Mann-Whitney test; ***P < 0.001.

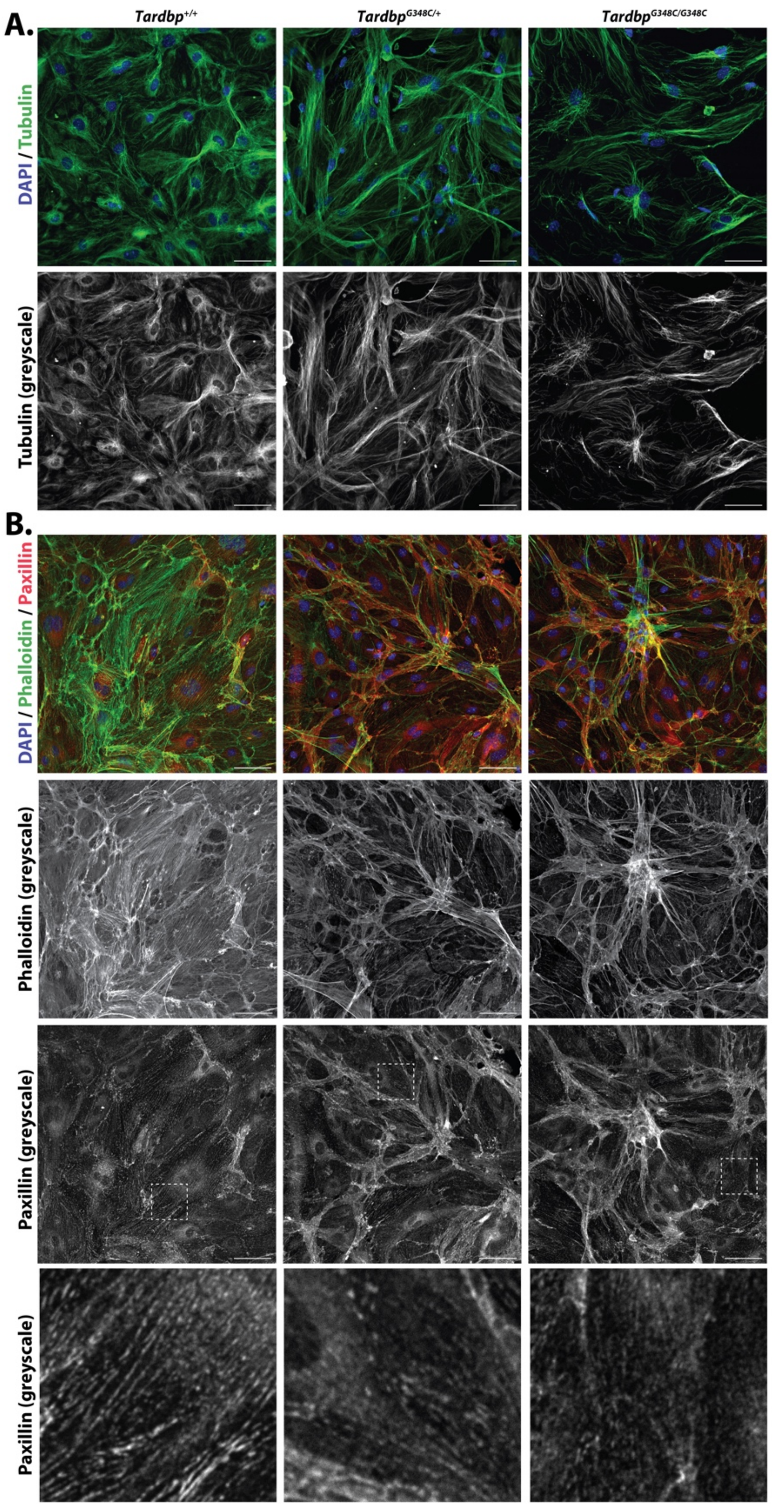

SI Figure 7. Cytoskeletal defects in endothelial cells of *Tardbp*$^{G348C/+}$ and *Tardbp*$^{G348C/G348C}$ mice. (A) Representative immunofluorescence images of DAPI and tubulin following isolation brain endothelial cells from the indicated genotypes. Multiple fields were taken from two mice of each genotype, littermate controls. (B) Immunofluorescence images of DAPI, phalloidin (actin) and paxillin (focal adhesions) in the isolated endothelial cells. Scale bars, 50 μm.

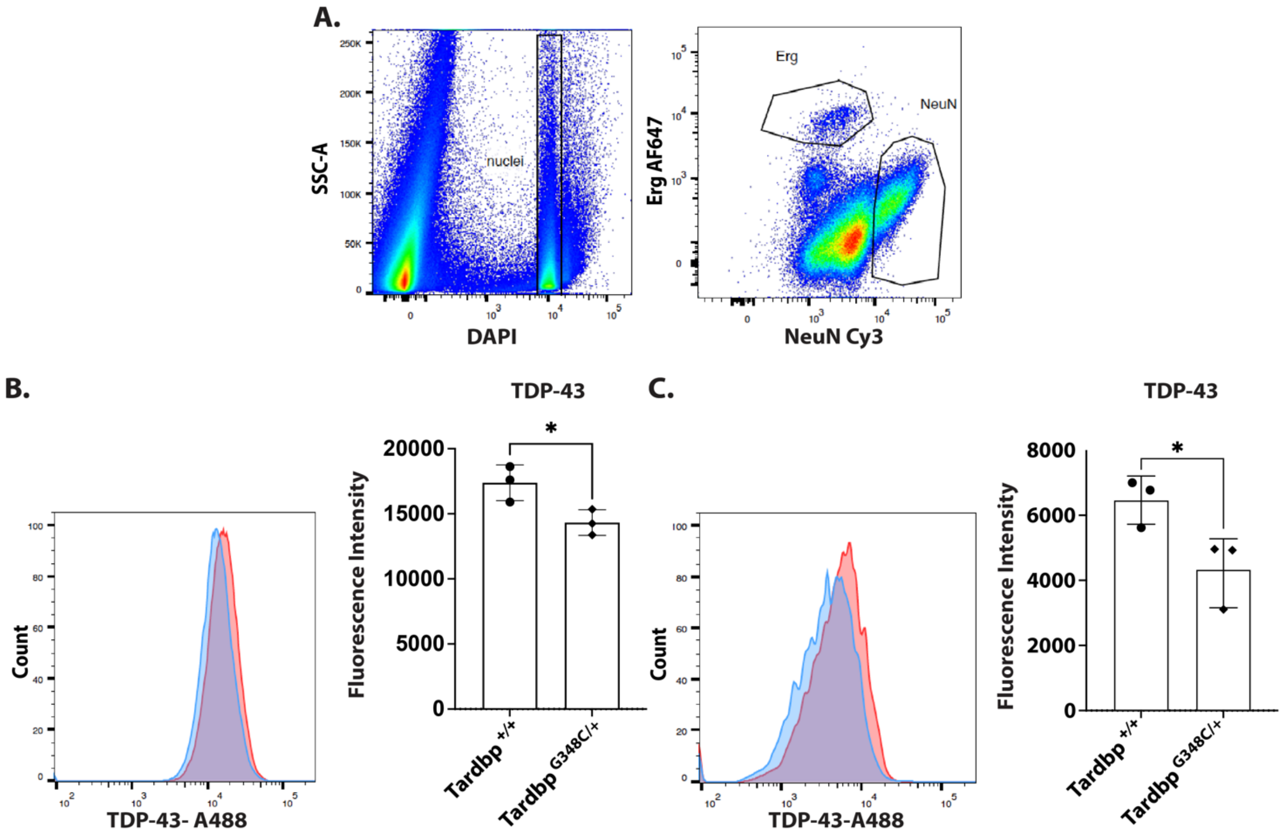

SI Figure 8. Quantitation of nuclear TDP-43 in *Tardbp*$^{G348C/+}$ neurons and endothelial cells. (A) Flow cytometry gating for Erg+DAPI+ endothelial nuclei and NeuN+DAPI+ neuronal nuclei. (B, C) Representative flow cytometry data plots and sample means for nuclear TDP-43 levels in NeuN+ neuronal nuclei (B) and Erg+ endothelial nuclei (C). Data are presented as means ± SEM. Statistical analysis was conducted using an unpaired Mann Whitney test, with significance levels as follows: *$P < 0.05$.

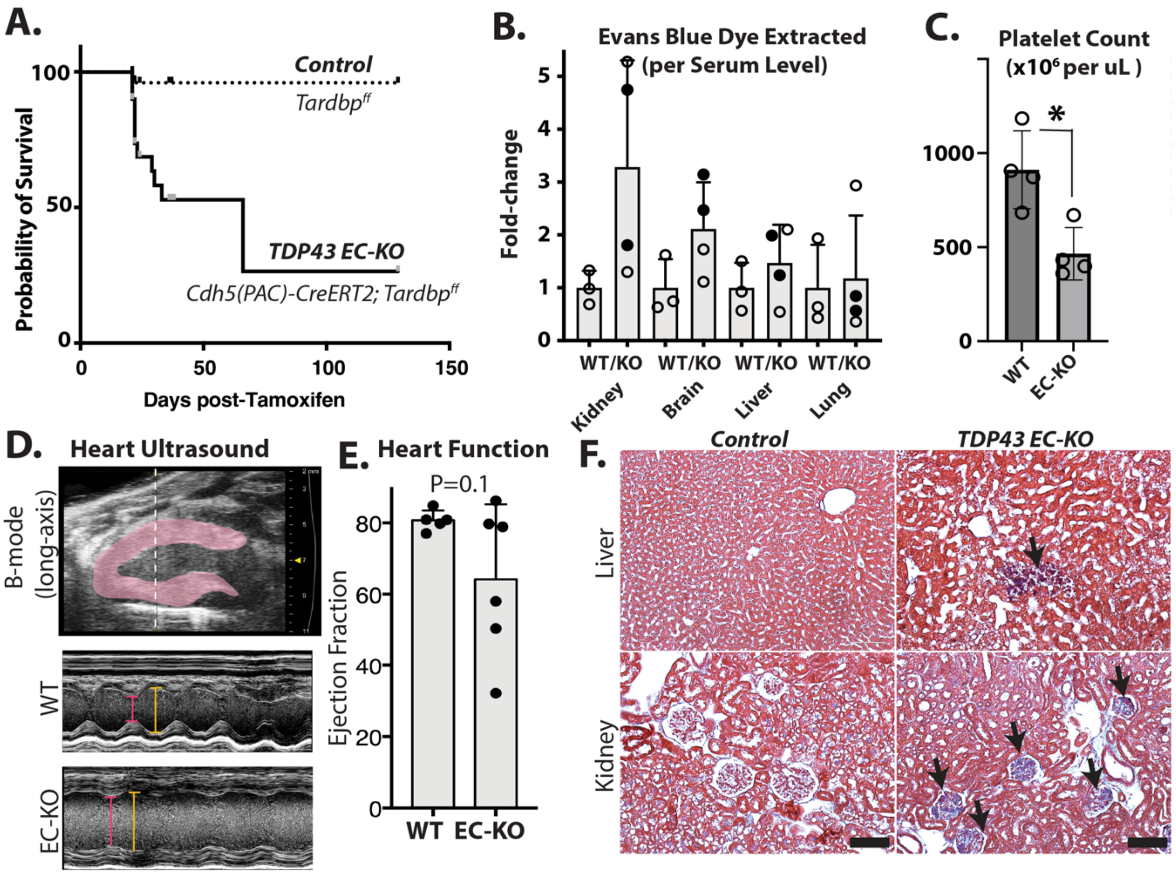

SI Figure 9. *Tardbp EC-KO* mice exhibit widespread endothelial dysfunction and vascular leak. Phenotypic analysis of TDP-43 EC-KO mice versus littermate controls at 3-4 weeks of age. (A) Mice are moribund by 3-4 weeks post-Tam treatment and gene excision (n=30 EC-KO and n=30 littermate controls at risk). (B) Vascular leak assessed by Evans blue (EB) dye extraction (tissue EB normalized by plasma EB, presented as a fold change over the same tissue from control mice). Dark dots indicate moribund mice at the time of tissue collection. (C) Absolute platelet count measured by flow cytometry of blood (CD41-FITC+), normalized to littermate controls. (D, E) Ultrasound with quantitation, demonstrating impaired ejection fraction in EC-KO mice (n=6 EC-KO and 5 littermate controls). (F) Fibrosis was observed in trichrome-stained tissues (blue staining indicates collagen). (C) Statistical analysis was performed using an unpaired two-tailed Student's t-test. (C&E) Statistical analysis was conducted using the Mann-Whitney test, *P<0.05.

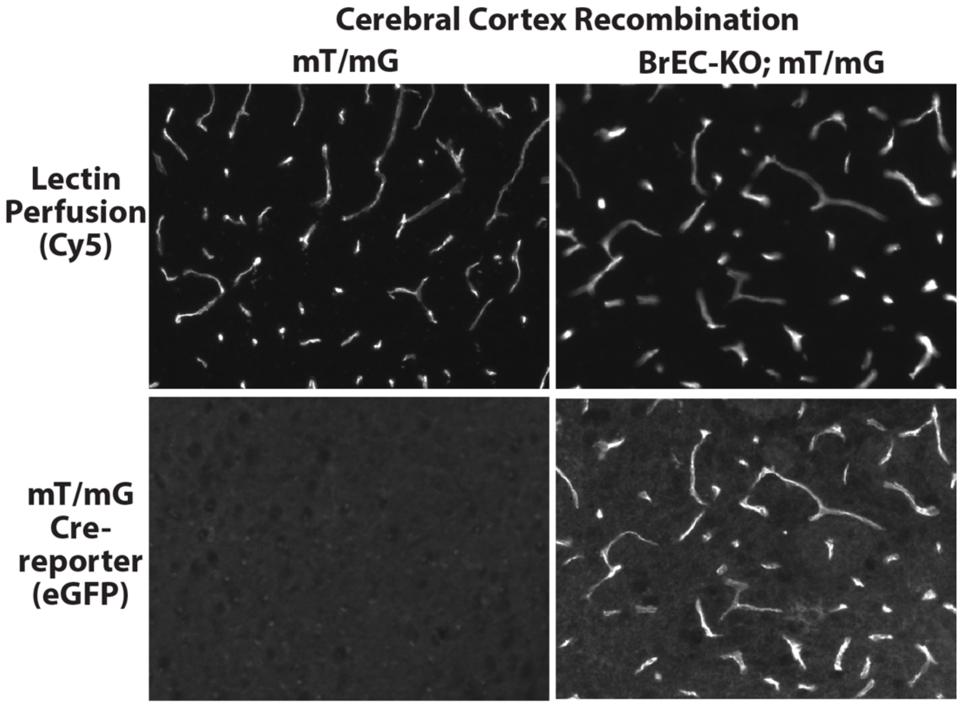

SI Figure 10. Slco1c1-CreERT2 is active in cortical vasculature. (A) BrEC-KO mice with mT/mG Cre reporter were treated with Tamoxifen and collected with Dylight649 lectin perfusion 12 weeks later. eGFP indicates Cre activity in cortical vessels.

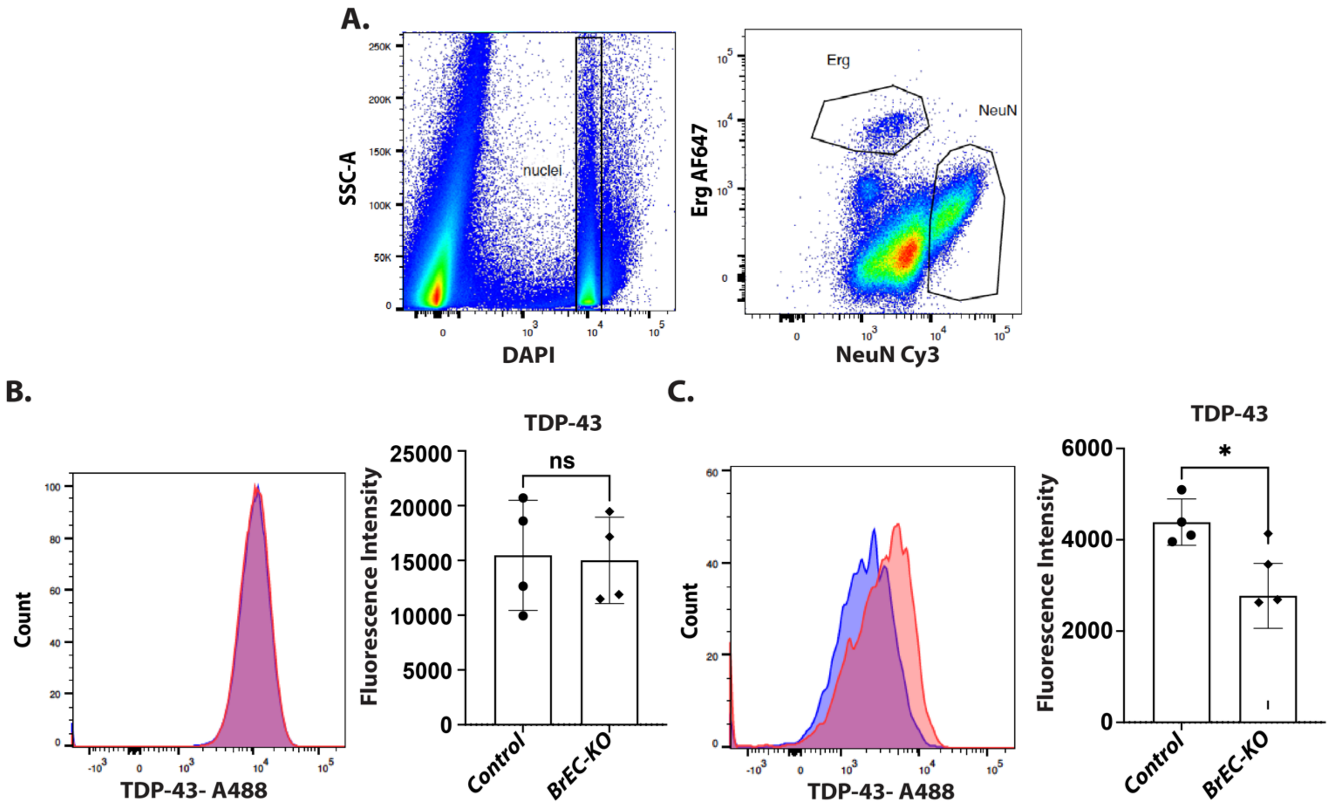

SI Figure 11. Quantitation of nuclear TDP-43 in BrEC-KO neurons and endothelial cells. (A) Flow cytometry gating for Erg+DAPI+ endothelial nuclei and NeuN+DAPI+ neuronal nuclei. (B, C) Representative flow cytometry data plots and sample means for nuclear TDP-43 levels in NeuN+ neuronal nuclei (B) and Erg+ endothelial nuclei (C). Data are presented as means ± SEM. Statistical analysis was performed using an unpaired Mann-Whitney test, with significance levels indicated as follows: ns (not significant), *$P < 0.05$.

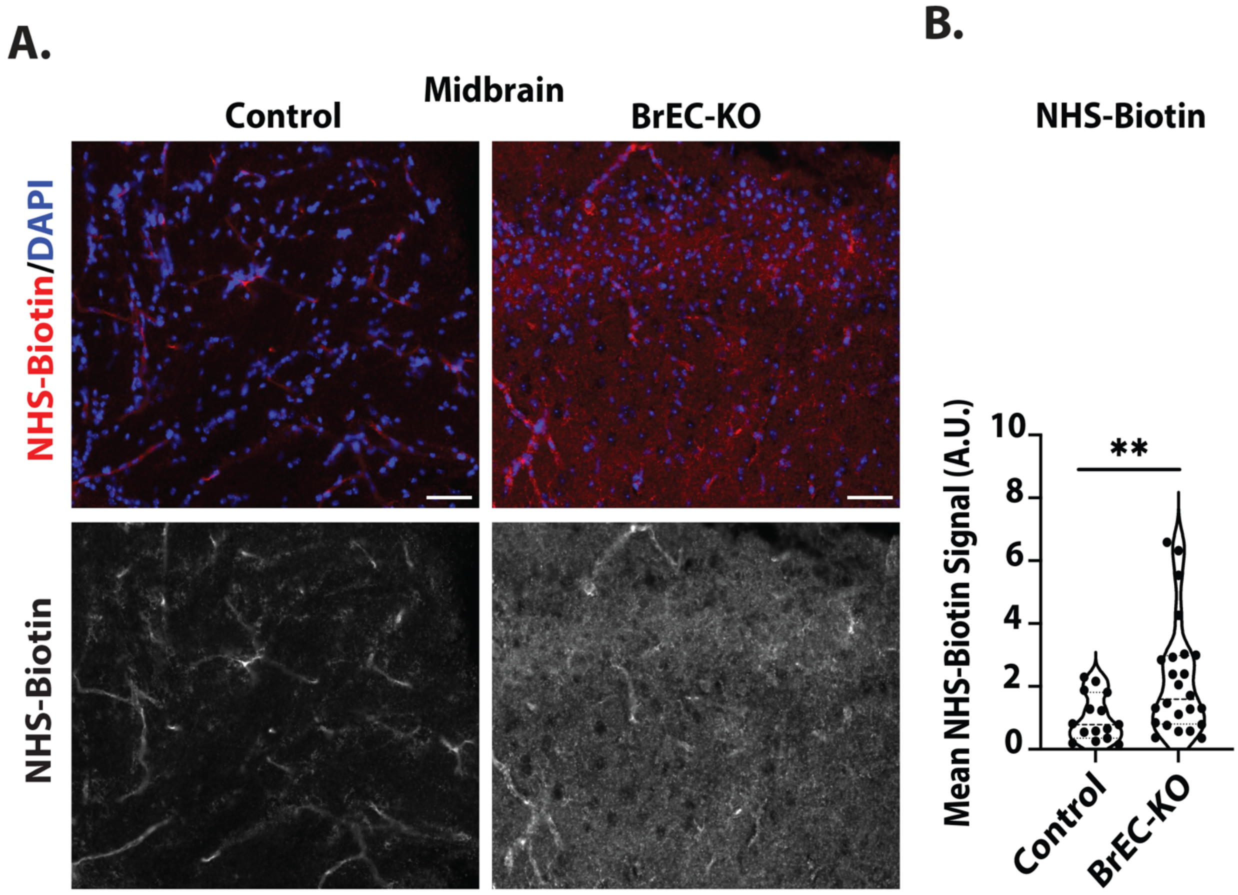

SI Figure 12. Blood-brain barrier disruption in *Tardbp* BrEC-KO, midbrain. (A) The representative immunofluorescence images of NHS-biotin leakage in mouse midbrain sections from 3-7-month-old mice (n=3 BrEC-KO and n=3 littermate controls) are shown. B) Quantification signal, with each data point representing the fluorescence image intensity from one image, multiple images per mouse. Scale bars, 50 µm. Data is presented as means ± SEM. Statistical analysis was performed using an unpaired two-tailed Mann Whitney test, with significance levels indicated as follows: **P<0.01.

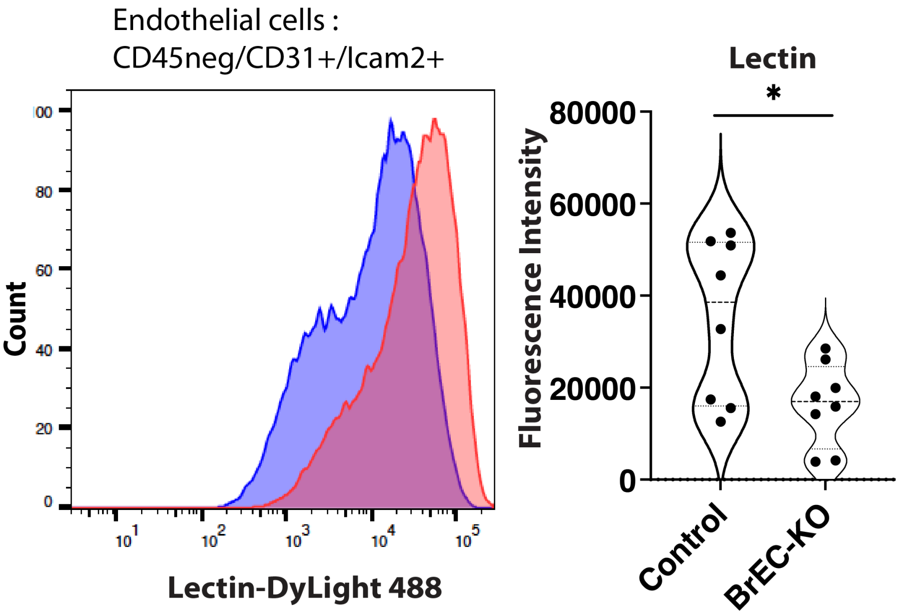

SI Figure 13. Tomato lectin staining of endothelium in BrEC-KO mice. Flow cytometry plot showing lectin+ staining intensity of sorted endothelial cells from BrEC-KO mice and littermate controls, and quantitation of mean lectin staining in sorted endothelial cells (N=8 and N=8). Scale bars, 50 µm. Data are presented as means ± SEM. Statistical analysis was conducted using an unpaired Mann Whitney test, *P<0.05.

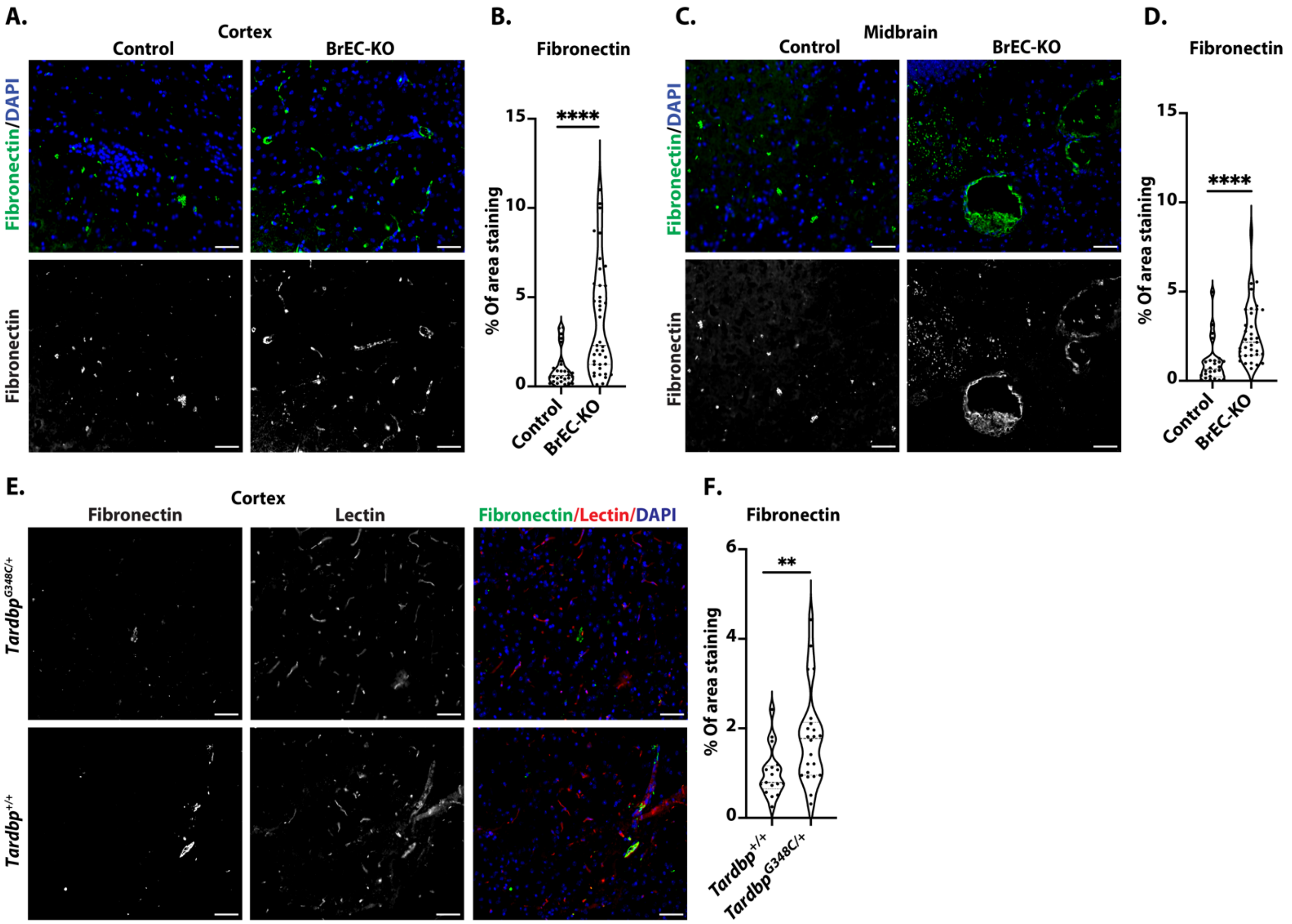

SI Figure 14. Increased vascular fibronectin in *Tardbp* BrEC-KO and *Tardbp*$^{G348C/+}$ mice. (A) Representative immunofluorescence images of fibronectin in mouse cortex and (C) midbrain sections from 8-11-month-old mice (n=3 BrEC-KO and n=3 littermate controls) are presented. Additionally, (E) representative immunofluorescence images of fibronectin in mouse cortex sections from 10-11-month-old mice (n=3 *Tardbp*$^{+/+}$ and n=3 *Tardbp*$^{G348C/+)}$ are shown. (B, D, F) Quantification signal, with each data point representing the fluorescence image intensity from one image, multiple images per mouse. Scale bars, 50 µm. Data is presented as means ± SEM. Statistical analysis was performed using an unpaired two-tailed Mann Whitney test, with significance levels indicated as follows: **P<0.01, ****P<0.0001.

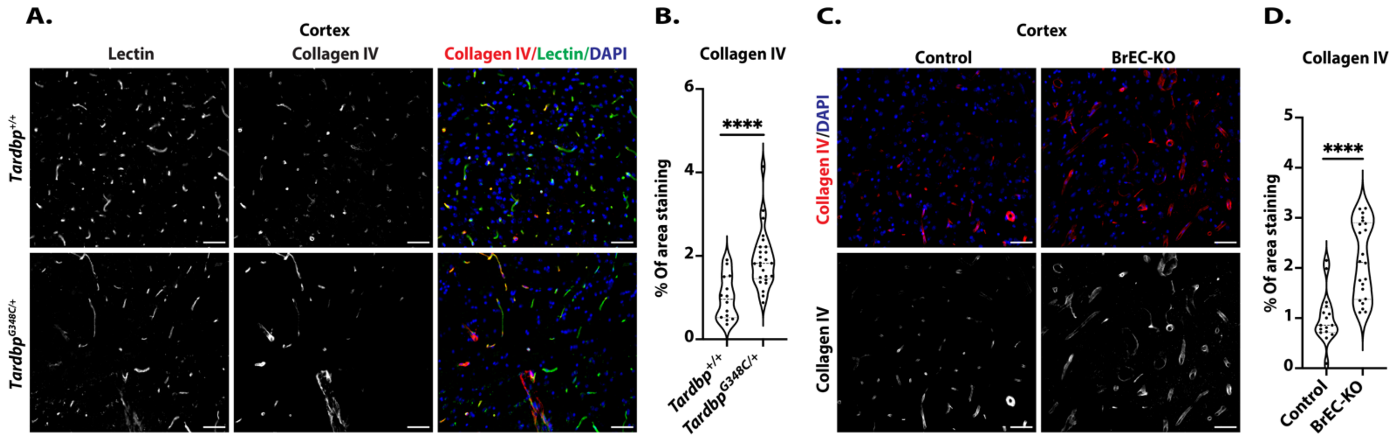

SI Figure 15. Increased Collagen IV expression in *Tardbp* BrEC-KO and *Tardbp*$^{G348C/+}$ mice. (A) Representative immunofluorescence images of Collagen IV in mouse cortex sections from 10-11-month-old mice (n=3 *Tardbp*$^{+/+}$ and n=3 *Tardbp*$^{G348C/+}$) are presented. Additionally, (C) representative immunofluorescence images of Collagen IV in mouse cortex sections from 8-11-month-old mice (n=3 BrEC-KO and n=3 littermate controls) are shown. (B&D) Quantification signal, with each data point representing the fluorescence image intensity from one image, multiple images per mouse. Scale bars, 50 µm. Data is presented as means ± SEM. Statistical analysis was performed using an unpaired two-tailed Mann Whitney test, with significance levels indicated as follows: ****P<0.0001

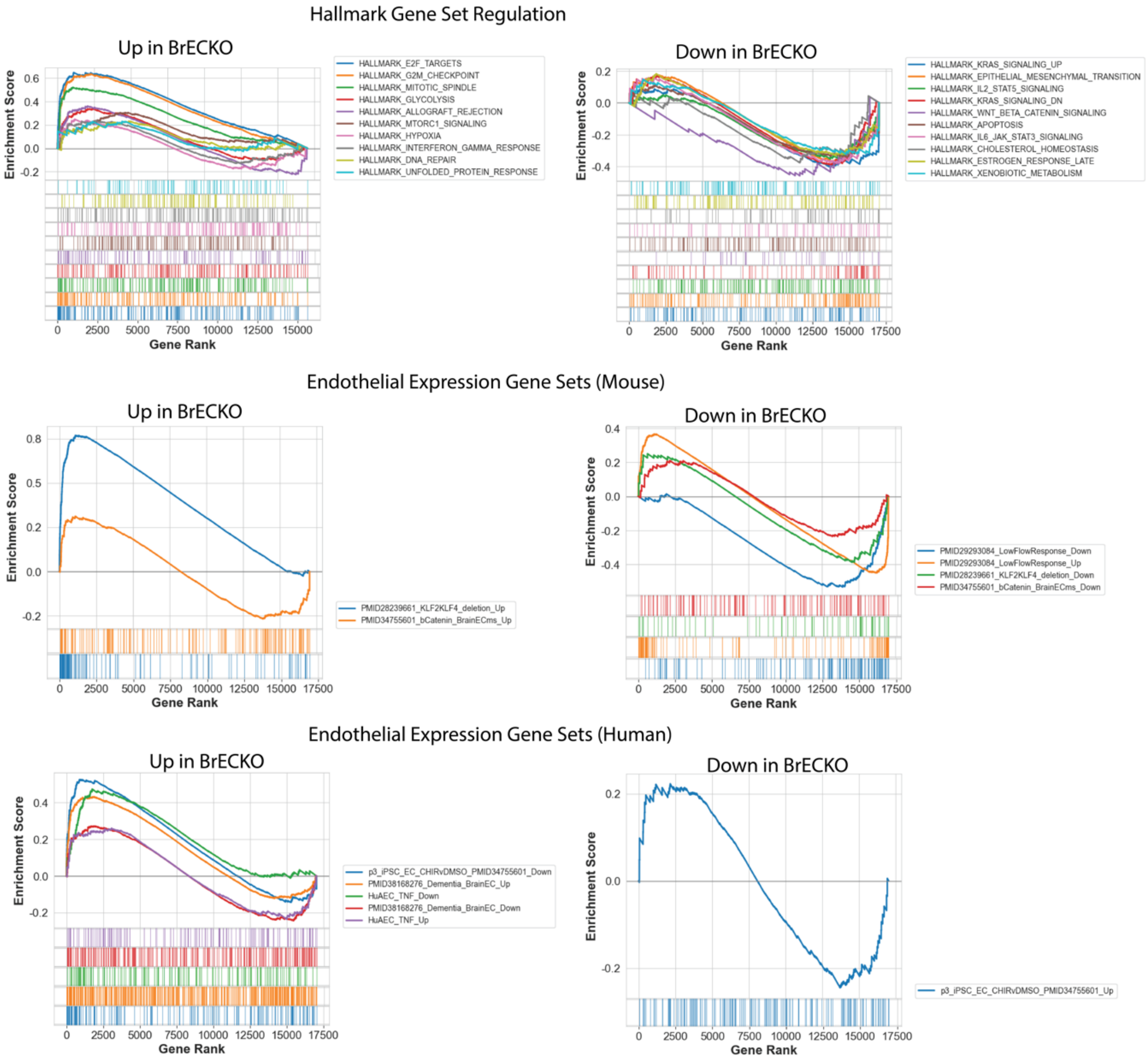

SI Figure 16. Gene Set Enrichment Analysis (GSEA) of BrEC-KO endothelial cells. GSEA analysis of differentially expressed genes (DESeq2, N=6 vs N=6) in endothelial cells sorted from BrEC-KO mice relative to littermate controls. Analysis by Hallmark genes and custom GSEA gene sets for endothelial specific responses, curated from literature and TNF-α stimulation of endothelial cells.

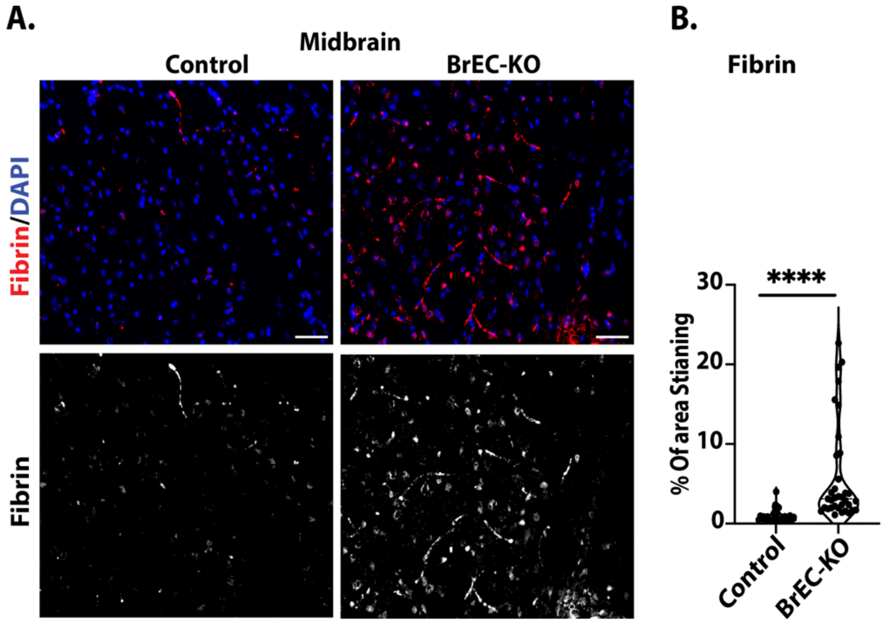

SI Figure 17. Fibrin deposition in BrEC-KO mouse midbrain. (A) Representative immunofluorescence images of fibrin deposition in mouse midbrain sections from 8-11-month-old mice (n=3 BrEC-KO and n=3 littermate controls) are shown. (B) Quantification signal, with each data point representing the fluorescence image intensity from one image, multiple images per mouse. Scale bars, 50 µm. Data is presented as means ± SEM. Statistical analysis was performed using an unpaired two-tailed Mann Whitney test, with significance levels indicated as follows: ****P<0.0001.

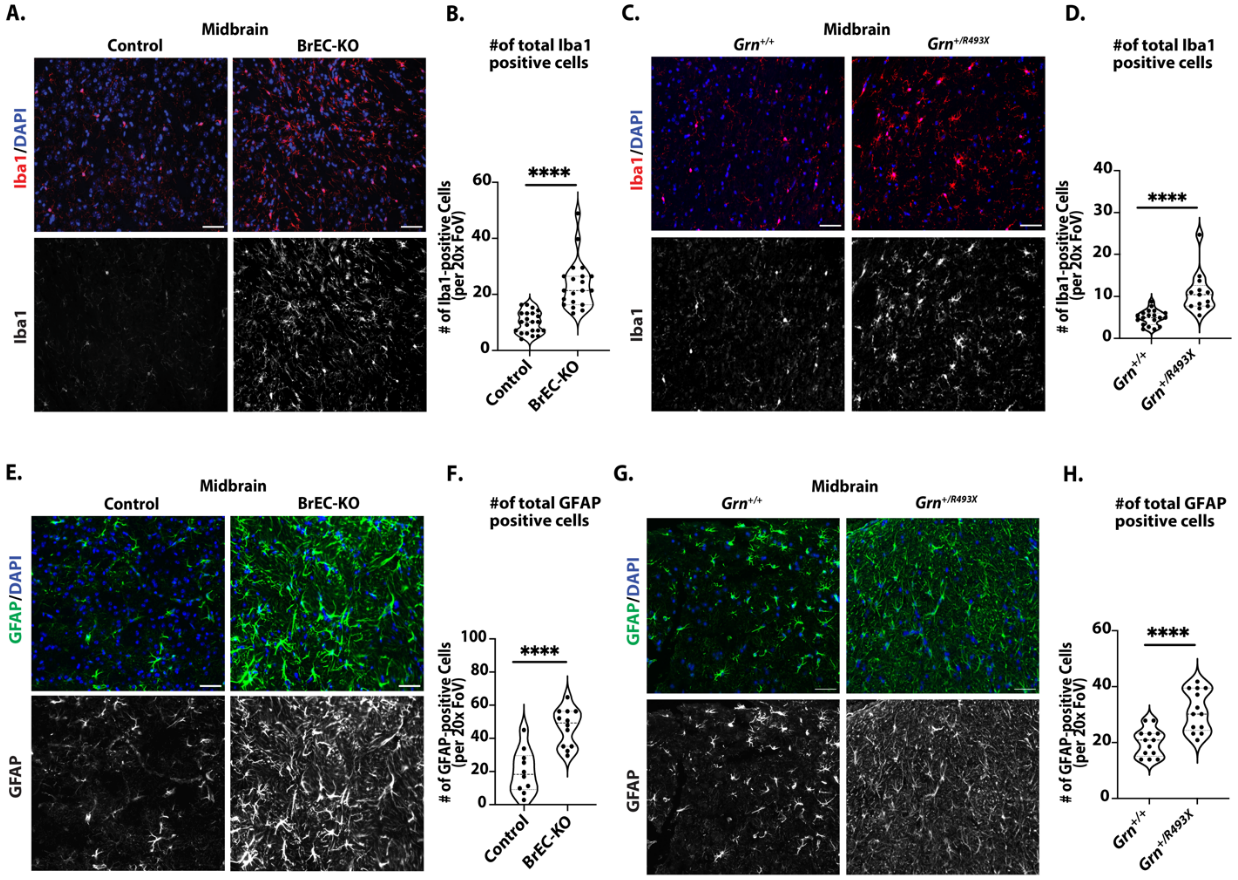

SI Figure 18. Assessment of astrocyte (GFAP) and microglia (Iba1) activation. (A, C) Representative immunofluorescence images of Iba1 staining of microglia in the mouse midbrain reveal consistent results across (n=3 BrEC-KO and littermate controls mice (n=3), as well as n=3 *Grn*$^{R493X/+}$ and littermate controls mice (n=3) and (E, G) GFAP staining of astrocytes reveals a substantial increase in astrocyte numbers, resembling astrogliosis observed in FTD. (B, D, F, H) Field of view (FoV) is 0.16mm^2. Quantification of data with each data point representing the number of activated cells in an image. multiple images per mouse. Scale bars, 50 µm. Data are presented as means ± SEM. Statistical analysis was conducted using an unpaired two-tailed Mann Whitney test, with significance levels indicated as follows: ****P<0.0001.

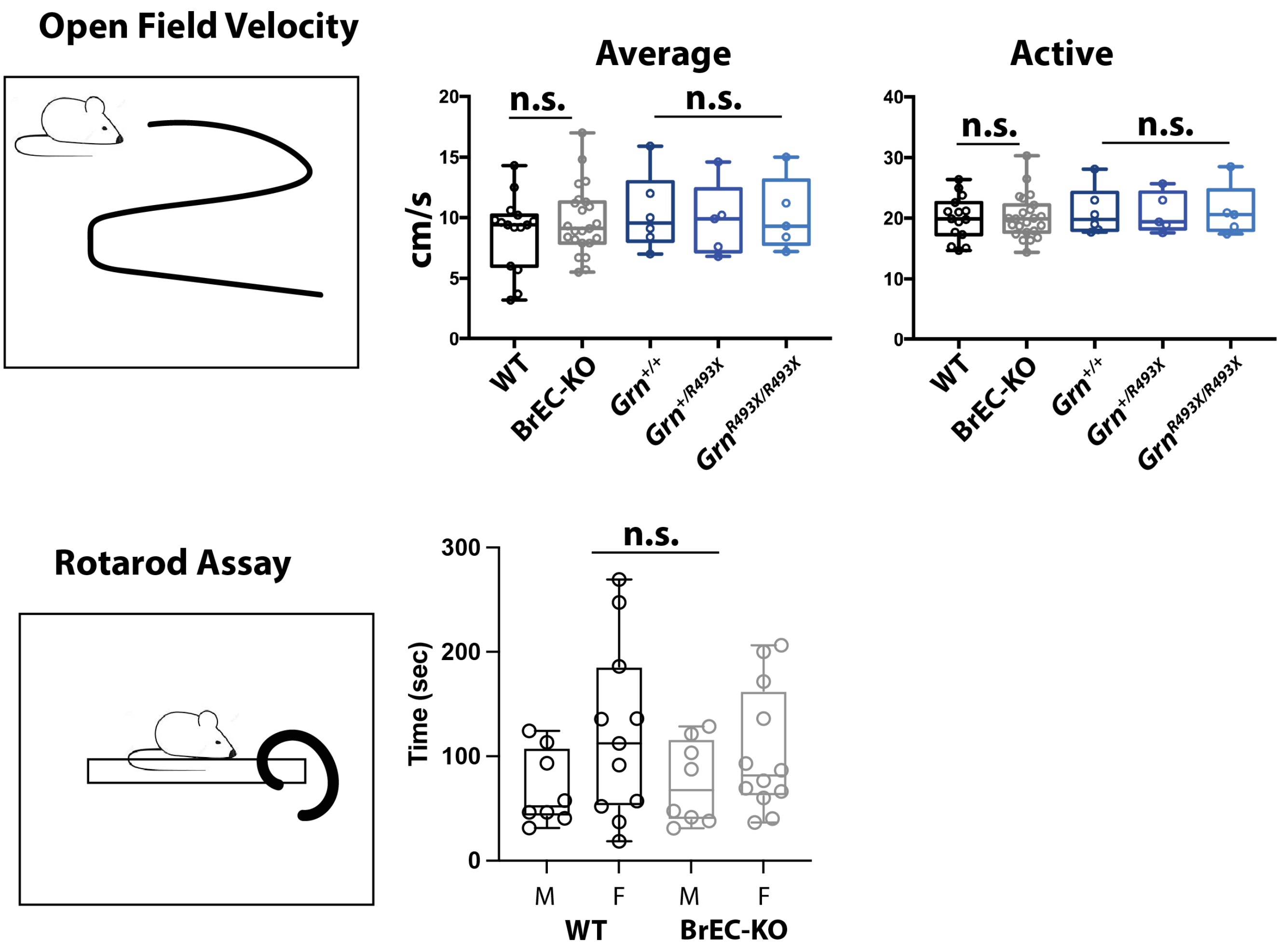

SI Figure 19. Open-field and rotarod assessment of BrEC-KO mice. BrEC-KO and littermate controls were assessed in behavioral core along with a knock-in mouse model of Frontal Temportal Lobe Dementia (FTD, $Grn^{+/R493X}$). No difference was observed in open field velocity.

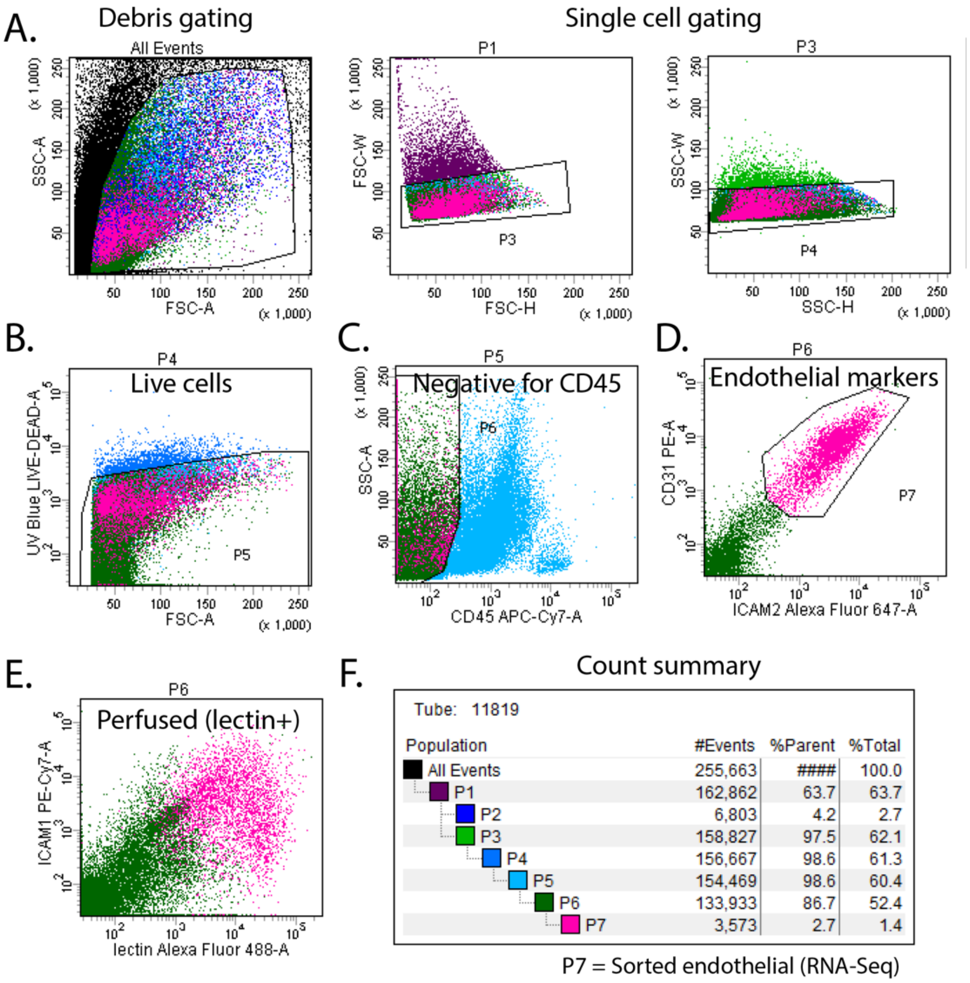

SI Figure 20. Representative sorting of endothelial cells for RNA-sequencing analysis. Example flow cytometry plots for the isolation of endothelial cells from brain tissue for RNA-sequencing analysis. (A) Gating out debris and isolating single endothelial cells by forward and side-scatter. (B) Removal of dead cells (Live-Dead dye+). (C) Isolation of CD45 negative cells to remove hematopoietic cells. (D) Sorting based on endothelial markers CD31 and Icam2. (E) Analysis of a third marker, from tomato lectin perfusion, showing that the sorted endothelial cells are lectin+ (and lining perfused vessels). (F) Typical count and percentages of endothelial cells isolated from brain tissue.

SI Table 1. CellSamples
Table with three sheets, describing a list of samples used for RNA-sequencing (“RNA_seq_samples”) and the comparisons used for DESeq2 analysis of differential expression by gene (“ComparisonsDESeq2”) and for Leafcutter analysis of splicing (“ComparisonsLeafcutter"). This data is used for the analysis presented in figures 5 and 6. Raw data has been deposited in NIH Sequence Read Archive, and processed count tables from RSEM used for DESeq2 analysis are provided in SI Table 2.

SI Table 2. GeneExpressionCountsRSEM
Table with three sheets, from RSEM analysis of STAR aligned BAM files, as outlined in materials and methods. The Posterior Mean Estimate (pme) for read counts is provided in the tables for the samples used for DESeq2 analysis. The DESeq2 results are provided in SI Table 3.

SI Table 3. DESeq2results
Table with 9 comparisons, one per sheets, as outlined in SI Table 1. Results are from DESeq2 analysis of samples and counts described in SI Table 2.

SI Table 4. GSEA
Table with weighted Gene Set Enrichment Analysis (GSEA) of ranked genes from DESeq2 results (SI Table 3) for Kegg, Hallmark and custom GSEA sets. Custom GSEA sets are described in SI Table 5.

SI Table 5. CustomGSEAsets
Table with two sheets, “Mouse” and “Human” describing the source of custom curated endothelial gene sets for the indicated responses. Data were derived from published papers (with PMID listed) or data from the lab on TNF treatment of human aortic endothelial cells, as described in SI Table 1.

SI Table 6. APAlyzerUTR
Table showing results of APAlyzer analysis of annotated alternative polyadenylation sequences (UTR), in human brain endothelial cells with and without suppression of TDP-43. Source data is described in SI Table 1.

SI Table 7. Leafcutter
Table showing results of Leafcutter analysis of alternative splicing in the comparisons outlines in SI Table 1.
